# CircTHBS1 drives gastric cancer progression by increasing INHBA mRNA expression and stability in a ceRNA- and RBP-dependent manner

**DOI:** 10.1038/s41419-022-04720-0

**Published:** 2022-03-25

**Authors:** Shengkui Qiu, Bowen Li, Yiwen Xia, Zhe Xuan, Zheng Li, Li Xie, Chao Gu, Jialun Lv, Chen Lu, Tianlu Jiang, Lang Fang, Penghui Xu, Jing Yang, Ying Li, Zetian Chen, Lu Zhang, Linjun Wang, Diancai Zhang, Hao Xu, Weizhi Wang, Zekuan Xu

**Affiliations:** 1 Department of General Surgery, The First Affiliated Hospital of Nanjing Medical University, Nanjing, 210029 Jiangsu Province China; 2 Department of General Surgery, The Second Affiliated Hospital of Nantong University, Nantong, 226001 Jiangsu Province China; 3 Jiangsu Key Lab of Cancer Biomarkers, Prevention and Treatment, Collaborative Innovation Center for Cancer Personalized Medicine, Nanjing Medical University, Nanjing, 211166 Jiangsu Province China

**Keywords:** Gastric cancer, Long non-coding RNAs

## Abstract

Circular RNAs (circRNAs) play vital regulatory roles in the progression of multiple cancers. In our study, transcriptome analysis and self-organizing maps (SOM) were applied to screen backbone circRNAs in gastric cancer (GC). Upon validation of the expression patterns of screened circRNAs, gain- and loss-of-function assays were performed in vitro and in vivo. Underlying mechanisms were investigated using RNA pull-down, luciferase reporter assay and RNA immunoprecipitation. The expression of circTHBS1 was significantly increased in GC and associated with poor prognosis. CircTHBS1 facilitated the malignant behavior and epithelial-to-mesenchymal transition of GC cells. Mechanistically, circTHBS1 sponged miR-204-5p to promote the expression of Inhibin Subunit Beta A (INHBA). Moreover, circTHBS1 could enhance the HuR-mediated mRNA stability of INHBA, which subsequently activated the TGF-β pathway. Our research identified circTHBS1 as an oncogenic circRNA that enhances GC malignancy by elevating INHBA expression, providing new insight and a feasible target for the diagnosis and treatment of GC.

## Introduction

As one of the most malignant tumors, gastric cancer (GC) ranks fourth in cancer-related deaths worldwide [1]. At present, surgery is the only potential curative option for GC patients [2]; however, the high recurrence rate and low 5-year survival rate remain great challenges for GC patients [3]. Therefore, novel biomarkers for early diagnosis and effective therapeutic targets are urgently needed.

In recent years, the critical role of non-coding RNAs (ncRNAs) in GC has received increasing attention [4–6]. In our previous study [7], a comprehensive miRNA sequencing was performed and a unique conserved miRNA signature has been identified in mouse and human GC. And a representative set of miRNAs were validated in more than 400 samples from 3 independent cohorts. However, long non-coding RNAs were not involved in this study. As a special type of long non-coding RNA with a covalently closed loop, circular RNA (circRNA) is generated from precursor mRNA transcripts via a back-splicing process [4]. It regulates tumor progression via multiple mechanisms, the most important means of which is serving as a competitive endogenous RNA (ceRNA); in this role, circRNAs weaken the inhibitory effects of miRNAs on their target genes [7–9]. However, the circRNA landscapes of GC and the comprehensive link between circRNAs and miRNAs have not been investigated clearly. Therefore, in this study, 24 paired of gastric samples previously used for miRNA sequencing were subjected to ceRNA arrays to profile circRNAs and mRNAs in GC.

Transcriptome sequencing is one of the most common methods to detect tumor regulatory targets [10]. High-throughput data with too many parameters will be generated after transcriptome sequencing [11], therefore, visualizing and filtering out functional targets in these complex data is a substantial challenge. As an artificial neural network algorithms, self-organizing map (SOM) transforms high-latitude data into a two-dimensional lattice map on which the geographic distance between nodes represents their mathematical similarity [12, 13]. It has been widely accepted in many applications, such as prediction of arthritis [14], analysis of spatial relationships of COVID-19 [15], building gene regulatory networks from scATAC-seq and scRNA-seq [16]. However, the application of SOM to construct a non-coding gene regulatory network for GC from ceRNA arrays has not been reported yet.

In this study, we aimed to elucidate the regulatory role of circRNAs in GC; thus, ceRNA arrays and SOM analysis were applied to screen backbone circRNAs. Then RNA pull-down, RIP, and mass spectrometry were used to reveal the underlying mechanisms. In conclusion, we proposed an essential circRNA target associated with GC progression and further explored its regulatory mechanism.

## Materials and methods

### Patient samples

A total of 96 pairs of cancerous and para-cancerous tissues from gastric cancer patients were collected from the Department of Gastric Surgery, the First Affiliated Hospital of Nanjing Medical University. These samples were collected successively from January 2015 to January 2019. Twenty-four pairs of this tissues were subjected to human ceRNA microarray (sample information was shown in Supplementary Table S4), and the remaining tissues were mainly used for qRT-PCR verification. The clinicopathological features of patients, which included age, sex, tumor location, tumor size, lymph node status, TNM stage (AJCC classification), histological differentiation grade was shown in Table 1. This study was evaluated and reviewed by the Ethics Committee of the First Affiliated Hospital of Nanjing Medical University. All patients mentioned above have agreed to participate and signed a written informed consent.

**Table 1:** Correlation between circTHBS1 expression and the clinicopathologic parameters in 72 GC patients.

Characteristic	Group	Case	Expression of circTHBS1		*p*-value
			High	Low	
**Age**	<60	26	15	11	0.326
	≥60	46	21	25	
**Gender**	Male	53	24	29	0.181
	Female	19	12	7	
**Location**	Upper 1/3	31	14	17	0.475
	Lower 2/3	41	22	19	
**T stage**	Tis-T2	19	5	14	0.016^*^
	T3-T4	53	31	22	
**Lymph node status**	Negative	26	8	18	0.014^*^
	Positive	46	28	18	
**TNM stage**	I–II	30	10	20	0.017^*^
	III–IV	42	26	16	
**Histological grade**	Low	51	31	20	0.004^*^
	Middle-high	21	5	16	
**All cases**		72			

* *p* < 0.05

### Microarray analysis

SurePrint G3 Human ceRNA Microarray (4×180 k) were applied to determine gene expression profile. The total RNA from 24 pairs tissues extracted with TRIzol reagent (Invitrogen, CA, USA) were subjected to quality inspection by NanoDrop ND-2000 spectrophotometer (Thermo Fisher, MA, USA). Then the labeling of circRNAs and mRNAs and the subsequent production of ceRNA microarrays were performed according to the standard operating procedures provided by Microarray itself. The US Agilent microarray scanner was employed for raw data acquisition and the GeneSpring software GX 11.0 was used for data normalization. The data of ceRNA microarrays were shown in supplementary Table S1 and 2.

### Cell culture

The HEK-293T cell line, human gastric epithelium cell line GES-1 and human GC cell lines AGS, HGC-27, KATOIII, MKN-45, NCI-N87 were purchased from the Cell Center of Shanghai Institutes for Biological Sciences. KATOIII was grown in Dulbecco’s Modified Eagle’s Medium (DME H-21 4.5 g/Liter Glucose). The culture medium for AGS was Nutrient Mixture F-12K. And GES-1, HGC-27, MKN-45 andNCI-N87 were cultured in RPMI 1640 (w/o Hepes). All the culture mediums were supplemented with 1% penicillin-streptomycin and 10% fetal bovine serum (FBS), and all cells were cultured in a humidified 5% CO_2_ incubator at 37°C.

### RNA extraction and quantitative real-time PCR (qRT-PCR)

TRIzol Reagent (Invitrogen, Carlsbad, CA, USA) was used to extract total RNA from cells and tissues, whose RNA quality were then detected by Nanodrop 2000. The RNA isolation and extraction from nucleus and cytoplasm using PARISTM Kit (Thermo Fisher, MA, USA). The cDNA from circRNAs and mRNA was reverse transcribed with PrimeScript RT Master Mix Kit (Takara, RR036A, Japan), and cDNA from miRNA was synthesized by New Poly(A) Tailing Kit (Thermo Fisher, MA, USA). The above cDNA then was subjected to qRT-PCR with FastStart Universal SYBR Green Master Kit (Roche, Mannheim, Germany) and detected by ABI PRISM 7900HT sequence detection system (Applied Biosystems, Waltham, MA, USA). U6 and β-actin were used to normalize the relative miRNA and circRNA/mRNA expression levels respectively. All the primers were purchased from RiboBio (Guangzhou, China) and their sequence are listed in supplementary Table S5.

### RNase R treatment

For RNase R digestion, 2 μg of total RNA was incubated with or without 3 U/μg RNase R (Epicentre Technologies, Madison, WI, USA) for 15 min at 37°C, Then the relative expression levels of circTHBS1 and THBS1 mRNA were detected by qRT-PCR.

### Actinomycin D treatment and RNA stability assay

To verify the stability of circTHBS1 and THBS1 mRNA, AGS and HGC-27 cells were incubated with 2 mg/ml actinomycin D (Sigma-Aldrich, St. Louis, MO, USA), then the total RNA was harvested at different times (0 h, 4 h, 8 h, 12 h, 16 h). The relative remaining circRNA or mRNA were detected by qRT-PCR. The stability assessment of INHBA mRNA was the same as that of circTHBS1.

### RNA fluorescence in situ hybridization (FISH)

The fluorescent probes of circTHBS1 (Cy3-labeled) and miR-204-5p (Fam-labeled) were synthesized by Servicebio (Wuhan, China), and their sequences are listed in Supplementary Table S5. The AGS cells were first fixed with 4% paraformaldehyde following by permeabilization with 0.25% Triton X-100. Then the cells were hybridized at 37°C overnight in reaction buffer with labeled probes specific to circTHBS1. Leica SP5 confocal microscope (Leica Micosystems, Wetzlar, Germany) were used to detect the signals and acquire images.

### Plasmids and siRNA transfection and lentiviral transduction

To construct circTHBS1 overexpression plasmid, human circTHBS1 cDNA was synthesized by PrimerSTAR Max DNA Polymerase Mix (Takara, RR036A, Japan) and subcloned into the GV486 vector (GeneChem, China). circTHBS1-specific small interference RNAs (siRNAs) and siRNAs for HuR, INHBA were chemically synthesized by RiboBio (Guangzhou, China). Besides, the Oligonucleotides, including miR-204-5p mimics, inhibitor and the matched NC were also synthesized by RiboBio. And their siRNA sequences are listed in Supplementary Table S5. All transfection operations are strictly in accordance with the Lipofectamine3000 manufacturer’s protocol. The lentivirus-circTHBS1 and lentivirus-sh-circTHBS1 were constructed by GeneChem. After constructing stable transfected cell lines, animal experiments were carried out in accordance with the manufacturer’s protocol.

### Colony formation assay

AGS and HGC-27 cells (500 cells per well) were seeded in 6-well plates for two weeks, and the culture medium were refreshed every 3-5 days. On the 14th day, colonies were fixed with 4% paraformaldehyde for 15 minutes and stained with 0.1% crystal violet for 30 minutes. The proliferation ability of GC cells was mainly evaluated by the number and size of clones.

### Transwell assay

The transwell chamber covered with or without Matrigel Mix (Corning, NY, USA) were used to detect the invasion and migration ability of GC cells respectively. AGS (3×10^4^) and HGC-27 cells (2×10^4^) suspending in 200 μl serum-free medium were cultured in upper chambers, and 600 μl 10% FBS-medium was filled in the lower chamber. After 2 days incubation, 4% formaldehyde and 0.1% crystal violet were used to fix and stain the cells covered in the bottom chamber respectively. After erasing the cells in the upper chamber, the remaining cells adhered to the bottom chambers were photographed at 100× and counted in 10 different random fields of view.

### Cell counting kit-8 (CCK8) assay

The GC cells were seeded into 96-well plates (2×10^3^ per well) with 100 μl culture medium. CCK-8 solution (Dojindo, Kumamoto, Japan) was added into the wells (10 μl per well) followed by incubation at 37°C for 2 h. Then the cell viability was monitored for 5 days by microplate reader (BioTek, Winooski, VT, USA) at the wavelength of 450 nm.

### 5-Ethynyl-2’deoxyuridine (EdU) assay

The EdU assay was performed according to the manufacturer’s instructions of the Cell-light^TM^ EdU DNA cell proliferation kit (RiboBio, Guangzhou, China). In short, AGS and HGC-27 cells (1×10^3^ per well) were seeded into 96-well plates. The next day, the GC cells were incubating with 50 μM EdU for 2 h followed by fixing with 4% paraformaldehyde and staining with Apollo Dye Solution. After staining the nucleic acids of GC cells with Hoechst 33342, an Olympus microscope (Olympus, Tokyo, Japan) was used for images acquisition.

### Protein extraction and Western blot

RIPA lysis buffer (Beyotime, Shanghai, China) supplemented with protease and phosphatase inhibitor (NCM Biotech, Suzhou, China) was sued for protein extraction. After denaturing the proteins in boiling water with SDS-PADE protein buffer (Beyotime, Shanghai, China), the proteins were separated by SDS-polyacrylamide gel electrophoresis and then transferred to polyvinylidene difluoride membranes (Thermo Fisher, MA, USA). After being blocked with 5% blocking buffer for 2 h and incubating with the primary antibodies overnight at 4°C, the PVDF membranes were immunoblotted with the secondary antibodies for 2 h. Finally, the images of the blots on the membranes were obtained from a BioSpectrum 600 Imaging System (Thermo Fisher, MA, USA). Information about the primary and secondary antibodies used in western blot are listed in Supplementary Table S6. The uncropped blots are shown in Supplementary Fig. S7 and Fig. S8.

### Immunohistochemistry (IHC) analysis and hematoxylin-eosin (HE) staining

The tumor tissues were fixed in 4% paraformaldehyde after leaving the body, and then embedded in paraffin. For IHC, sections (5μm-thick) were incubated with the primary antibody overnight in a 4°C refrigerator and then incubated with secondary antibodies at room temperature for 1 h. After stained with chromogen (Servicebio, Wuhan, China), the images of the sections were acquired for examination. For HE staining, a hematoxylin-eosin staining kit (Beyotime, Shanghai, China) was used to stain the nucleus and cytoplasm directly. Information about the primary and secondary antibodies used in IHC and HE are listed in Supplementary Table S6.

### Immunofluorescence (IF) assay

3 × 10^4^ GC cells were seeded in the confocal dishes (Solarbio, Beijing, China) on the first day. The next day, the cells were fixed and permeabilized followed by blocking for 2 h. Then the cells were sequentially incubated with primary antibodies overnight and secondary antibodies for 1 h. Finally, the nuclei of cells were stained with DAPI, and LSM 710 confocal microscope (Zeiss, Germany) was used for Image acquisition. Information about the primary and secondary antibodies used in IF are listed in Supplementary Table S6.

### Dual luciferase reporter gene assay

Wild-type and mutant (mut-circTHBS1 or mut-INHBA) circTHBS1 and INHBA sequences were constructed and inserted into downstream of the luciferase reporter gene in the pmiR-RB-Report ™ vector (RiboBio, Guangzhou, China). AGS and HGC-27 cells (3 × 10^4^ per well) were seeded in 96-well plates. The next day the GC cells were co-transfected with the mimics of candidate miRNAs and negative controls and with the wild-type or mut-circTHBS1 reporter vector. 48 h latter, the luciferase activities (the ration of the firefly luciferase versus Renilla luciferase, LUC/RLUC) were detected by Dual-Luciferase Reporter Assay System (Promega, Madison, WI, USA).

### RNA pulldown and mass spectrometry

Magnetic RNA-protein Pull-down Kit (Thermo Fisher, MA, USA) was used to performer circRNA pulldown. The sequences of 3’ biotin-labeled probes (RiboBio, Guangzhou, China) targeting junction site of circTHBS1 are: GTCCAGCTCTACATTCGTAT-/3bio and AGTCCAGCTCTACATTCGTA-/3bio. Total RNA from circTHBS1-overexpressing HGC-27 cells (1.5×10^7^) were separately incubated with 100 nmol circRNA-specific probes and NC probes at 70°C for 5 min. Then the RNA mixture was incubated with Streptavidin Magnetic Beads at room temperature for 30 min. After washing away the unbound RNA with 20 mM Tris, the above beads were incubated with 100 total proteins at 4°Crotation. An hour and a half later, we washed away the unbound proteins and collected the RNA binding proteins with 50 μl elution buffer. Finally, the supernatant was subjected to silver stain and mass spectrum (OE Biotechnology, Shanghai, China). The same procedure was used for INHBA 3’UTR biotin-labeled probes (RiboBio, Guangzhou, China) to determine the interaction of INHBA 3’UTR with HuR.

### RNA-protein immunoprecipitation (RIP)

RIP assay was performed with the Magna RIP RNA Binding Protein Immunoprecipitation Kit (Millipore, MA, USA). The lysates of AGS and HGC-27 cells were incubated with anti-AgO2, anti-HuR, and IgG antibody-coated beads with rotation at 4°C overnight. Then, the beads were eluted with a RNeasy MinElute Cleanup Kit (Qiagen, Valencia, CA, USA) to extract the RNA complex. The RNA enrichment degree on probes was detected by reversing the above RNA complex followed by qRT-PCR. Information about the primary and secondary antibodies used in IF are listed in Supplementary Table 6.

### Animal experiments

The protocols of the animal experiments involved in this study were reviewed and approved by the Animal Ethics Committee of Nanjing Medical University.

The HGC-27 cells transfected with lentivirus were used to construct xenograft tumor models. 24 BALB/c nude mice (4-week-old) were randomly divided into four groups, then HGC-27 cells (1×10^7^ in 150 μl PBS) were injected subcutaneously into the forelimb underarms of each mouse. The volume (V = length×width^2^×0.5) of xenograft tumors was measure every week. The mice were sacrificed 4 weeks later, then the subcutaneous tumors were harvested, weighed, and stained with Ki67.

For the in vivo bioluminescence imaging, 24 mice (BALB/c nude, 5-week-old) were randomly divided into four groups (LV-sh-NC, LV-sh-circ, LV-Vector, LV-circ) to receive tail vein injection with the HGC-27 cells (1×10^6^). Four weeks later, the lung metastases of these mice were examined by bioluminescence imaging. After treatment with 2% isoflurane for anesthesia, D-luciferin sodium salt stock solution (150 mg/ml) was injected into the abdominal cavity of mice. Then, the anaesthetized mice were placed on the IVIS SpectrumD Xenogen Imaging System (Caliper Life Sciences, Hopkinton, MA, USA) for bioluminescent signals measurement. After IVIS, all the mice were sacrificed, and their lungs were harvested for H&E staining to further evaluate the lung metastasis.

### Statistical analysis

Quantitative data were recorded as mean value ± standard deviation, and all the experiments were repeated more than 3 times. Student’s t test was used to analyze the statistical difference between two groups, and analysis of variance (ANOVA) was applied to evaluate the differences among multiple groups. In addition, the relationship between circTHBS1 and clinical parameters was analyzed with Chi-square test. All statistical analyses were performed on SPSS software, version 26.0 (IBM, SPSS, Chicago, IL, USA) and GraphPad Prism, version 8.00 (GraphPad Software, La Jolla, CA, USA). *P* < 0.05 was statistically significant.

## Results

### ceRNA arrays and SOM analysis identified circTHBS1 as a key regulator in GC

**Fig. 1:**
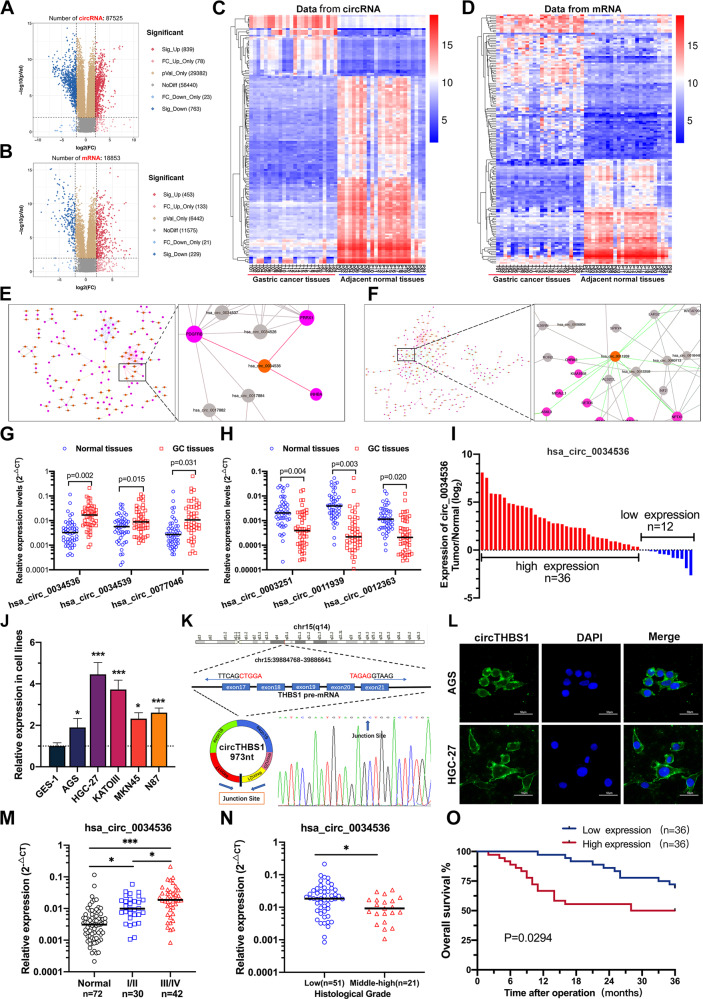
ceRNA arrays and SOM analysis identified circTHBS1 as a key regulator in GC. **A**, **B** The volcano plots showed the expression variations of circRNAs and mRNAs in 24 GC tissues compared to matched adjacent normal tissues. **C, D** Clustered heatmap of the top 150 de-regulated circRNAs and mRNAs from ceRNA arrays (Pearson correlation test). **E** Regulatory network formed by 73 upregulated circRNAs and their downstream mRNAs. **F** Regulatory network formed by 112 downregulated circRNAs and their downstream mRNAs. **G, H** The relative expression of the top 3 upregulated and 3 downregulated circRNAs (obtained from SOM analysis) in 48 paired tissues were detected by qRT-PCR. **I** Fold changes (log_2_) of has_circ_0034536 in each paired sample were arranged from high to low. **J** Expression levels of has_circ_0034536 in GES-1 and 5 gastric cancer cell lines. **K** The structure of circTHBS1 validated by Sanger sequencing. **L** Fluorescence in situ hybridization for circTHBS1. Nuclei were stained with DAPI. Scale bar = 50 μm. **M** The association of circTHBS1 expression and TNM stage. **N** The association of circTHBS1 expression and tumor histological grade. **O** Survival analysis of GC patients according to circTHBS1 expression. Quantitative data were presented as the mean ± SD. *P < 0.05, **P < 0.01, ***P < 0.001 (Student’s *t*-test).

To explore the role of ceRNA network in GC progression, 24 paired GC tissues and para-cancerous tissues were subjected to SBC human ceRNA arrays. A total of 1,602 differentially expressed circRNAs were identified (*p* < 0.01 and log_2_FC > 2.0), including 839 upregulated and 763 downregulated circRNAs in GC tissues (Fig. 1A). In addition, 453 significantly upregulated and 229 downregulated mRNA were detected in GC tissues compared to para-cancerous tissues (Fig. 1B). Then the heat maps of the top 150 de-regulate circRNAs and mRNAs were created to show their global expression changes (Fig. 1C, D). Next, Self-organizing map (SOM) analysis was applied to explore the potential circRNA-mRNA regulatory axis (supplementary Fig. S1A, B). Eventually, 185 differentially expressed circRNAs were screened, including 73 upregulated and 112 downregulated circRNAs (Fig. 1E, F). The top 3 significantly upregulated and 3 downregulated circRNAs were further verified by qRT-PCR in 48 paired GC tissues and para-cancerous tissues (Fig. 1G, H). We found that the expression of hsa_circ_0034536 (termed circTHBS1) was upregulated in GC tissues with the most significant difference (Fig. 1I). Meanwhile, higher expression levels of circTHBS1 were detected in GC cell lines compared to the gastric mucosal epithelial cell lines (GES-1) (Fig. 1J). Then the junction site and circular structure of circTHBS1 were successfully detected by Sanger sequencing and agarose gel electrophoresis assays (Fig. 1K and Supplementary Fig. S2A). Moreover, fluorescence in situ hybridization (FISH) combined with qRT-PCR analysis revealed that circTHBS1 was mainly located in the cytoplasm (Fig. 1L and Supplementary Fig. S2B). In addition, treatment with actinomycin D and RNase R showed that circTHBS1 exhibited a longer transcriptional half-time and enhanced stability compared to linear THBS1 (Supplementary Fig. S2C-F). The expression of circTHBS1 and patients’ clinicopathological parameters are shown in Table 1. Tumor size, lymphatic metastasis, AJCC TNM stage, and histological grade were positively correlated with the expression of circTHBS1 (Fig. 1M, N). Moreover, high circTHBS1 expression in the GC tissues contributed to poor prognosis (Fig. 1O, *p* = 0.0249), suggesting that circTHBS1 could be a promise diagnostic biomarker in GC.

### CircTHBS1 promotes the proliferation, migration, and invasion of GC cells

**Fig. 2:**
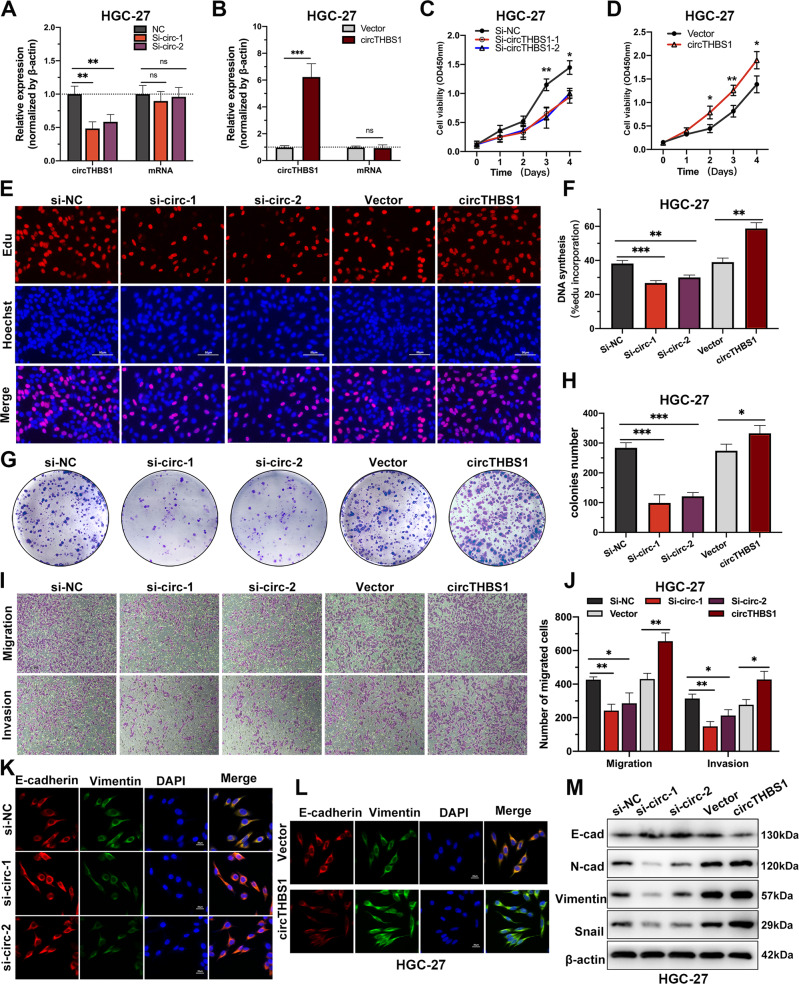
CircTHBS1 promotes GC cells proliferation, migration, and invasion in vitro. **A, B** The relative expression of circTHBS1 and THBS1 mRNA in AGS and HGC-27 cells transfected with circTHBS1 overexpression plasmids or siRNA. **C, D** The growth curves of HGC-27 cells evaluated by CCK-8 assays after knockdown or overexpression of circTHBS1. **E**, **F** The Effect of circTHBS1 on the proliferation of HGC-27 cells detected by EdU assays. Scale bar =50 μm. **G**, **H** Colony formation assay were used to detect the effect of circTHBS1 expression on the proliferation of HGC-27 cells. **I, J** Transwell assays in HGC-27 cells with knockdown or overexpression of circTHBS1 to detect migration and invasion ability. **K, L** The expression of E-cadherin (red) and vimentin (green) in HGC-27 cells detected by immunofluorescence staining, and DAPI (blue) was applied for nuclear staining. Original magnification, 400×; scale bar=20 μm. **M** Expression of EMT-related proteins in HGC-27 cells were detected by Western blot; β-actin was used as an internal control. Quantitative data presented as the mean ± SD. *P < 0.05, **P < 0.01, ***P < 0.001 (Student’s *t*-test).

To investigate the biological functions of circTHBS1, two specific small interfering RNAs (siRNAs, supplementary Fig. S3A) and overexpression plasmids were constructed. As expected, the siRNAs and overexpression plasmid affected the expression of circTHBS1, but not the linear THBS1 mRNA (Fig. 2A, B). Then CCK-8 assays showed that circTHBS1 knockdown significantly suppressed the proliferation of AGS and HGC-27 cells compared to the controls (Fig. 2C and supplementary Fig. S3B); whereas circTHBS1 overexpression promoted the proliferation of GC cells (Fig. 2D and supplementary Fig. S3C). Similarly, the pro-proliferative effect of circTHBS1 was observed by EdU assays and colony formation assays (Fig. 2E–H and supplementary Fig. S3D–G). Then, transwell assays revealed that knockdown of circTHBS1 inhibited the migration and invasion of AGS and HGC-27 cells compared to controls; while overexpression of circTHBS1 led to the opposite effects (Fig. 2I, J and supplementary Fig. S3H, I). In addition, the immunofluorescence assays showed that the expression of E-cadherin was increased, and expression of Vimentin was decreased after circTHBS1 knockdown. And converse results were acquired after circTHBS1 overexpression (Fig. 2K, L and supplementary Fig. S3J, K). Consistently, the results of western blot revealed that circTHBS1 overexpression promoted the expression of N-cadherin, Snail, and Vimentin, but downregulated the E-cadherin expression in AGS and HGC-27 cells (Fig. 2M and supplementary Fig. S3L). Taken together, these findings suggested that circTHBS1 promoted the proliferation and EMT of GC cells in vitro.

### CircTHBS1 activates the TGF-β pathway via regulation of INHBA

**Fig. 3:**
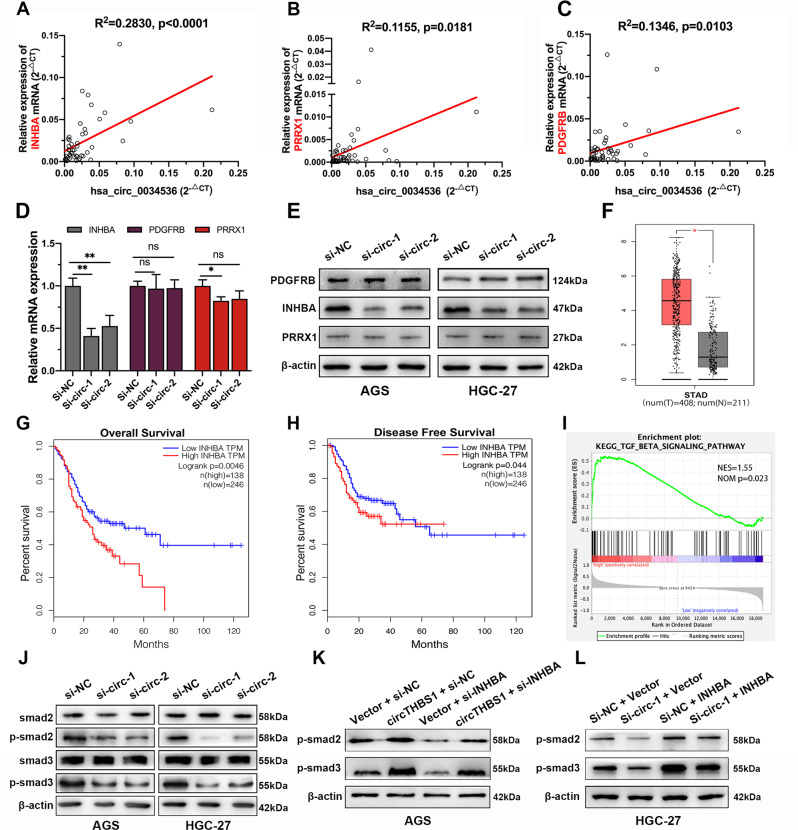
circTHBS1 activates TGF-β signaling pathway via upregulation of INHBA. **A**–**C** The correlation of circTHBS1 with its downstream target genes were verified by qRT-PCR in 48 GC tissues. These three genes were the most strongly correlated with circTHBS1 expression after SOM analysis. **D** Expression of INHBA, PDGFRB, and PRRX1 in HGC-27 with knockdown of circTHBS1 were detected by qRT-PCR. **E** Western blot of INHBA, PDGFRB, and PRRX1 in GC cells transfected with si-circTHBS1. β-actin was used as an internal control. **F** The expression of INHBA in GC tissues and normal tissues (from the TCGA database). **G, H** The overall survival and disease-free survival of GC patients based on INHBA expression in TCGA database. **I** Gene enrichment analysis (GSEA) based on the results of our ceRNA array. Briefly, the expression of circTHBS1 from ceRNA arrays was used as a phenotypic label and the data of all the mRNAs was defined as gene set. **J** Western blot of key proteins involved in TGF-β pathway in AGS and HGC-27 cells with knockdown of circTHBS1. **K** Western blot assay showed that knockdown of INHBA in AGS cells impaired the activation of TGF-β pathway by overexpression of circTHBS1. **L** Overexpression of INHBA in HGC-27 cells rescued the inhibitory effect of Knockdown circTHBS1 on TGF-β pathway. Quantitative data presented as the mean ± SD. *P < 0.05, **P < 0.01, ***P < 0.001 (Student’s *t*-test).

To validate the results of SOM analysis that the expression of INHBA, PRRX1, and PDGFRB were correlated with circTHBS1, qRT-PCR was performed, and the results showed that INHBA was most significantly correlated with circTHBS1 (Fig. 3A–C). Moreover, both qRT-PCR and western blot results in AGS and HGC-27 cells showed that silencing circTHBS1 only reduced the expression of INHBA compared to controls (Fig. 3D, E). In addition, data from the TCGA database showed that INHBA was highly expressed in GC tissues and positively correlated with poor prognosis (Fig. 3F–H). To further explore the potential downstream regulatory mechanisms of circTHBS1, a gene set enrichment analysis was performed. We found that higher circTHBS1 expression was significantly positively correlated with the regulation of TGF-β signaling pathway (*p* = 0.023, Fig. 3I). The results from western blot confirmed that p-smad2, p-smad3 were significantly downregulated after circTHBS1 knockdown in AGS and HGC-27 cells (Fig. 3J). Moreover, the effect of knockdown or overexpression of circTHBS1 on the TGF-β pathway could be rescued by overexpression and knockdown of INHBA, respectively (Fig. 3K, L). The above results suggest that circTHBS1 promotes GC progression via targeting INHBA and thus activating the TGF-β pathway.

### CircTHBS1 acts as a miRNA sponge for miR-204-5p to upregulate INHBA expression

**Fig. 4:**
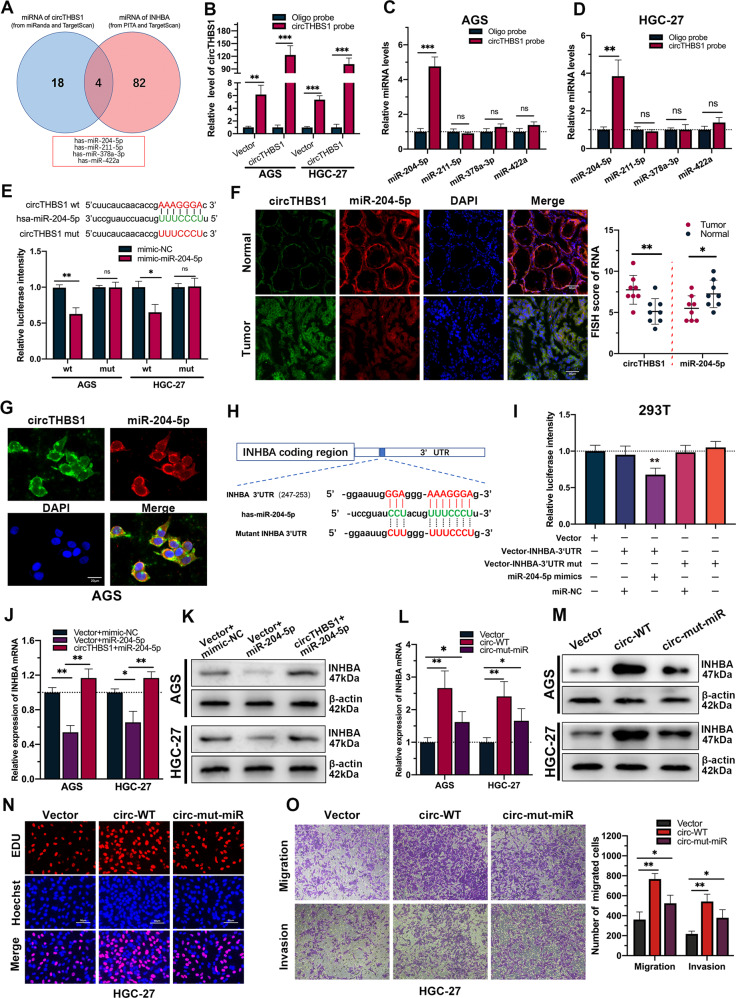
CircTHBS1 acts as a miRNA sponge of miR-204-5p to upregulate INHBA expression. **A** Venn diagram showing the overlap of miRNAs sharing the same miRNA response elements (MREs) between circTHBS1 and INHBA. Briefly, miRNAs for circTHBS1 were predicted according to the TargetScan (http://www.targetscan.org) and miRanda (http://www.microrna.org) databases; upstream miRNAs for INHBA were predicted from the TargetScan and PITA databases (http://genie.weizmann.ac.il/pubs/mir07/mir07_dyn_data.html). **B** The circRNA-specific probes effectively pulled down and enriched circTHBS1. **C, D** The relative enrichment of four candidate miRNAs pulled down by circTHBS1 probe or oligo probe in AGS and HGC-27 cells. **E** Schematic diagram of the predicted binding sites between miR-204-5p and circTHBS1 (top). Luciferase reporter assays verified that miR-204-5p physically binds to circTHBS1 (bottom). **F, G** FISH for circTHBS1 (green) and miR-204-5p (red) in GC tissues and matched normal tissues (*n* = 8) and GC cells (AGS cell line). DAPI were used to stained for nuclei (blue). Scale bars are 50μm and 20μm respectively. **H** The schematic diagram of the binding sites between miR-204-5p and wild-type or mutant (mut) INHBA 3’UTR. **I** Luciferase reporter assays used to confirm the combination between miR-204-5p and INHBA 3’UTR. **J, K** The relative expression of INHBA was detected by qRT-PCR and western blot in AGS and HGC-27 cells transfected with control vector, miR-204-5p mimic or circTHBS1 overexpression plasmids. **L, M** Expression levels of INHBA in AGS and HGC-27 cells transfected with different types of circTHBS1 overexpression plasmids. These plasmids included empty plasmid (Vector), wild-type plasmid (circ-WT), and plasmids in which the binding site of miR-24-5p was mutated (circ-mut-miR). **N, O** Colony formation and Transwell assays of HGC-27 transfected with vector, circ-WT, or circ-mut-miR overexpression plasmids. Quantitative data of 3 independent experiments were presented as the mean ± SD. *P < 0.05, **P < 0.01, ***P < 0.001 (Student’s *t*-test).

To explore the potential relationship between circTHBS1 and INHBA, bioinformatic analysis was performed. The results showed that circTHBS1 and INHBA shared miRNA response elements (MREs) in miR-204-5p, miR-211-5p, miR-378a-3p, and miR-422a (Fig. 4A). We first performed RIP with anti-AGO2 in AGS cells and found that endogenous circTHBS1 was significantly immunoprecipitated, indicating that circTHBS1 acted as a miRNA sponge (Supplementary Fig. S4A). Then, biotin-labeled circTHBS1 probes were designed, and RNA pull-down assays followed by qRT-PCR were performed in AGS and HGC-27 cell lysates (Fig. 4B). The results showed that only miR-204-5p could be captured by the circTHBS1 probes (Fig. 4C, D). Subsequent luciferase reporter assays in GC cells further confirmed that circTHBS1 physically combine with miR-204-5p (Fig. 4E). Additionally, significant cytoplasmic colocalization of circTHBS1 and miR-204-5p was detected by FISH in GC tissues and AGS cells. (Fig. 4F, G). Taken together, our data indicated that circTHBS1 serves as a sponge for miR-204-5p. In our previous study [7], miR-204-5p has been confirmed to be downregulated in GC, here we found that overexpression of miR-204-5p significantly inhibited the proliferation, migration, and invasion of AGS and HGC-27 cells (Supplementary Fig. S4B–D). To validate whether miR-204-5p directly targets INHBA mRNA, the miRNA target site in the 3’-UTR of INHBA was mutated (Fig. 4H) and dual-luciferase reporter assays were performed. The results showed that miR-204-5p could bind to the 3’UTR region of INHBA (Fig. 4I). Moreover, overexpression of circTHBS1 in AGS and HGC-27 cells reversed miR-204-5p-induced low expression of INHBA (Fig. 4J, K), further confirming that circTHBS1 could regulate the expression of INHBA by sponging miR-204-5p. Interestingly, we transferred plasmids with the miR-204-5p binding site mutated (circ-mut-miR) into AGS and HGC-27 cells and found that these plasmids still partially promoted INHBA expression compared to the empty vectors (Fig. 4L, M). Moreover, EdU assays and Transwell assays showed that the circ-mut-miR plasmids partially promoted the proliferation, migration, and invasion of AGS and HGC-27 cells (Fig. 4N, O and Supplementary Fig. S4E, F). Based on the above findings, we speculated that circTHBS1 may regulate INHBA expression through other mechanisms in addition to ceRNA.

### CircTHBS1 stabilizes INHBA mRNA by interacting with HuR and promoting its cytoplasmic export

**Fig. 5:**
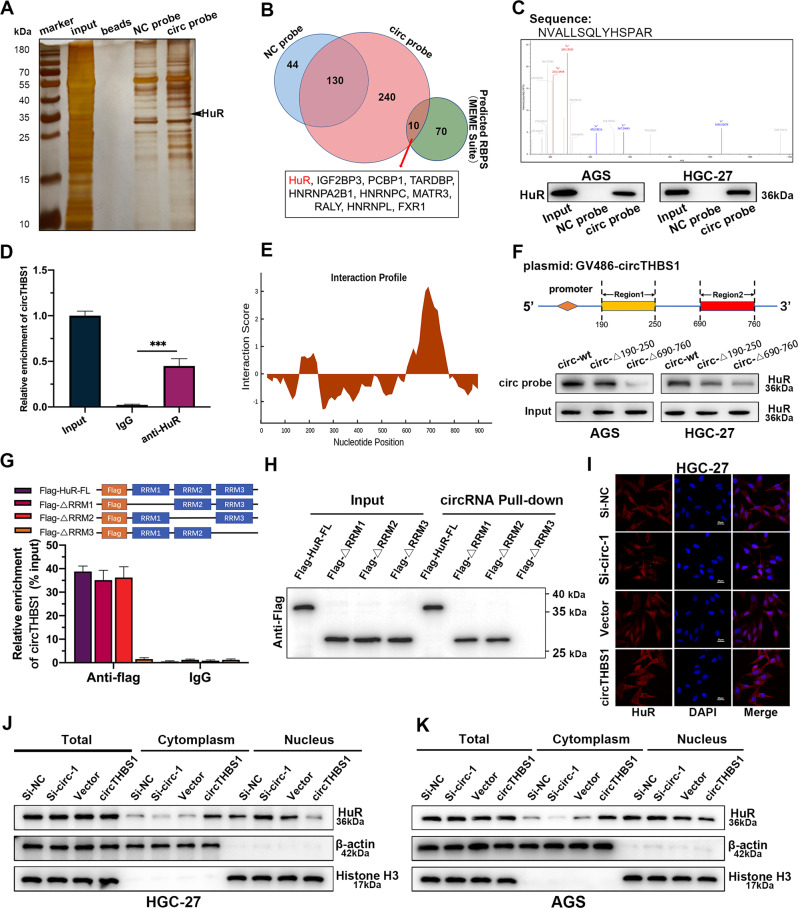
CircTHBS1 interacts with HuR and promotes its cytoplasmic transport. **A** Silver staining of circTHBS1 pulldown. **B** Venn diagram showing the target RBPs of circTHBS1 from mass spectrometry and prediction of MEME suite. **C** The secondary mass spectrometry of HuR protein (top). Western blot verified that HuR was enriched in the circTHBS1 probe (bottom). **D** RIP assay shown that HuR protein precipitated with circTHBS1 in GC cell lysates. **E** The predicted binding regions of circTHBS1 with HuR. **F** The schematic diagram of the truncated fragment of circTHBS1 overexpression plasmids (top). After GC cells were transfected with wild-type or truncated circTHBS1 overexpression plasmids, RNA pull-down was performed with circTHBS1 specific probes (bottom). **G** The full-length or truncated forms of flag-labeled recombinant HuR protein were incubated with HGC-27 cell lysates, then the recovered circTHBS1 levels were detected by qRT-PCR. **H** RNA pull-down experiments were performed by using circTHBS1-specific probes against full-length or truncated forms of flag-tagged recombinant HuR proteins. **I** Immunofluorescent of HuR (red) in HGC-27 cells after knockdown or overexpressing circTHBS1. DAPI (blue) for nuclear staining. Original magnification=400×; scale bar=20 μm. **J, K** The expression levels of HuR in total lysates or subcellular fractions were detected by western blotting. AGS and HGC-27 cells were transfected with si-NC, si-circTHBS1, vector, and circTHBS1 overexpression plasmids. Quantitative data were presented as the mean ± SD. *P < 0.05, **P < 0.01, ***P < 0.001 (Student’s *t*-test).

**Fig. 6:**
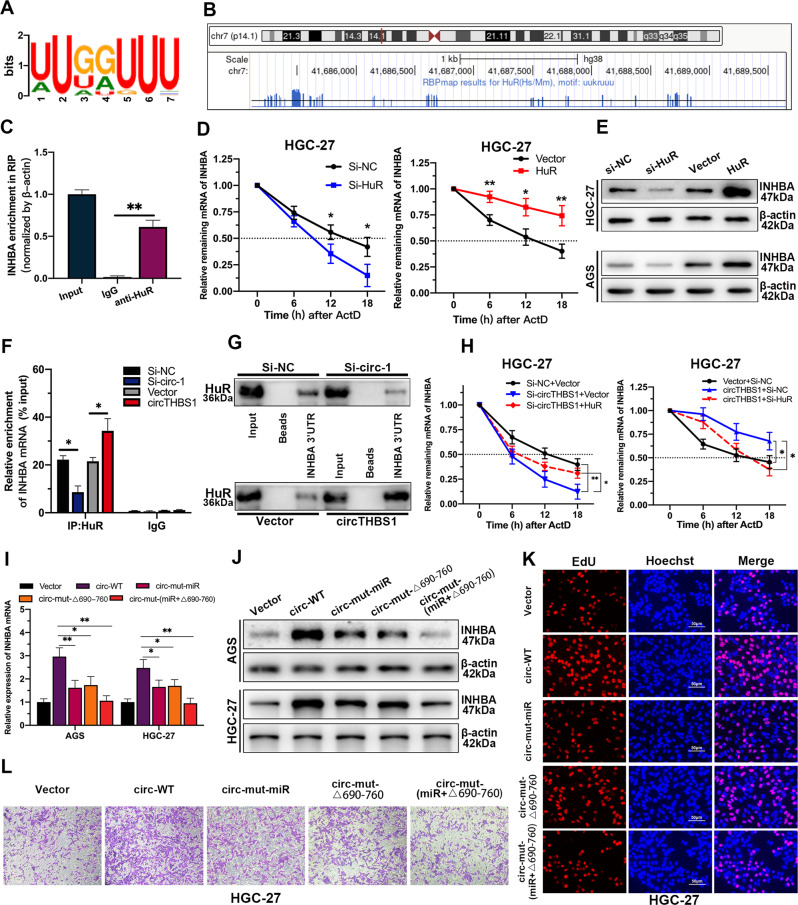
CircTHBS1 stabilizes INHBA mRNA via interacting with HuR. **A** The motif of HuR. **B** The HuR binding sites in INHBA 3’UTR region predicted by RBPmap. The bottom blue vertical lines represented the Hub binding sites in 3’UTR of INHBA mRNA. **C** RIP assay shown that HuR protein interacted with INHBA mRNA. **D** Degradation rates of INHBA mRNA were detected by qRT-PCR in HuR overexpressing or knockdown HGC-27 cells at different time periods. **E** The expression of INHBA protein in GC cells transfected with si-NC, si-HuR, vector or HuR overexpression plasmids was detected by Western blot. **F** RIP assays showed the content of INHBA mRNA co-precipitated with HuR after knockdown or overexpression of circTHBS1 in HGC-27 cells. **G** RNA pulldown assays were performed in HGC-27 cells with biotin-labeled INHBA 3’UTR. The HGC-27 cells were transfected with si-circTHBS1 or circTHBS1 overexpression plasmids. **H** Degradation rate of INHBA mRNA in HGC-27 cells transfected with different plasmids or small interferences. **I, J** The expression of INHBA in GC cells transfected with wild-type and mutant circTHBS1 overexpression plasmids. circ-mut-miR indicated the miR-204-5p binding site was mutant, circ-mut-Δ690-760 referred to the sequence from 690 to 760 of circTHBS1 was truncated. circ-mut-(miR + Δ690-760) referred to both mutated miRNA binding site and truncated part of the sequence. **K, L** EdU assays and Transwell of HGC-27 cells treated with wild-type or mutant circTHBS1 overexpression plasmids. Scale bar=50 μm. Quantitative data presented as the mean ± SD. *P < 0.05, **P < 0.01, ***P < 0.001 (Student’s *t*-test).

In explaining the phenomenon that circTHBS1 plasmids with mutated miR-204-5p binding site still possessed partial cancer-promoting function, RNA binding proteins (RBPs) caught our attention. This is because interaction with RBPs is another essential method for circRNA to regulate expression of downstream genes. To verify our hypothesis, RNA pull-down assays followed by SDS-PAGE and mass spectrometry were performed in HGC-27 cells (Fig. 5A). A total of 10 RBPs were found to potentially interact with circTHBS1 according to the results of mass spectrometry analysis and predictions from the MEME suite databases (https://meme-suite.org/) (Fig. 5B). Among them, HuR scored the highest in the mass spectrometry analysis and thus was selected for further study. Then the interaction between circTHBS1 and HuR was confirmed by RNA pull-down and RIP assays. As the results showed, HuR proteins in AGS and HGC-27 cell lysates were pulled down by the circTHBS1 probes (Fig. 5C), and circTHBS1 were also precipitated by the anti-HuR antibody (Fig. 5D). According to the CatRAPID (http://service.tartaglialab.com) database, the two regions where circTHBS1 binds to HuR are located at 190-250 nt and 690-760 nt, respectively (Fig. 5E). Subsequently, circTHBS1 plasmids with these two regions truncated were constructed and transfected into AGS and HGC-27 cells. RNA pull-down assays showed that when the 690-760 nt region of circTHBS1 was truncated, the HuR protein captured by the circRNA probes was significantly reduced, indicating that circTHBS1 mainly interacts with HuR in this region (Fig. 5F). To further clarify which structural domain of HuR contributes to its interaction with circTHBS1, HuR mutants with truncated individual protein structural domains were constructed. RIP assays revealed that RNA recognition motif 3 (RRM3) of HuR specifically bound to circTHBS1 (Fig. 5G). Then RNA pull-down in 293 T cells showed that the HuR protein with the RRM3 region truncated could not be pulled down by circTHBS1 probes (Fig. 5H). As a nucleoplasmic shuttle protein, the subcellular localization of HuR was shown to perform different biological functions [17]. In our study, we found that circTHBS1 overexpression promoted the export of HuR protein from the nucleus to the cytoplasm, whereas circTHBS1 knockdown reduced the cytoplasmic level of HuR (Fig. 5J, K). Consistently, immunofluorescence confirmed that circTHBS1 expression was positively correlated with the content of HuR in the cytoplasm (Fig. 5I and supplementary Fig. S5A, B). As reported, one important function of cytoplasmic HuR is to stabilize target mRNAs by binding to U-or AU-rich RNA stretches (AREs) in their 3’-untranslated region (UTR) [18]. And the INHBA mRNA possess a long U- and AU-rich 3’UTR containing several predicted HuR-binding motifs (Fig. 6A, B). As a result, our RIP assays showed that INHBA mRNA was significantly enriched in anti-HuR compared to IgG (Fig. 6C). Moreover, actinomycin D assays in GC cells revealed that HuR knockdown reduced the levels of INHBA mRNA and shortened its transcript half-life compared to controls, whereas the reverse results were observed when circTHBS1 was overexpressed (Fig. 6D, E and supplementary Fig. S5C, D). To investigate whether circTHBS1 is involved in regulating the interaction between HuR and INHBA, we performed RIP assays in HGC-27 cells and found that circTHBS1 knockdown decreased the binding of INHBA mRNA to HuR, while overexpression of circTHBS1 increased the level of INHBA mRNA captured by HuR (Fig. 6F). Consistently, the RNA pulldown assays showed that knockdown or overexpression of circTHBS1 decreased or increased the enrichment of HuR protein bounding to the 3’ UTR of INHBA mRNA, respectively (Fig. 6G). Rescue experiments demonstrated that the decrease in INHBA mRNA stability caused by knockdown of circTHBS1 in HGC-27 and AGS cells was reversed after HuR overexpression. Conversely, the increase in INHBA mRNA stability induced by circTHBS1 overexpression was reversed after HuR silencing (Fig. 6H and supplementary Fig. S5E, F). To investigate whether circTHBS1 exerts its bio-function in GC cells through simultaneous regulation of HuR and miR-204-5p, circTHBS1 plasmids with mutant miR-204-5p binding site (circ-mut-miR), truncated HuR binding region (circ-mut-Δ690-760), and both mutations (circ-mut-miR + Δ690-760) were transfected into AGS and HGC-27 cells respectively. The results showed that circ-mut-miR and circ-mut-Δ690-760 still partially promoted INHBA expression compared with empty vector, but circ-mut-miR + Δ690-760 nearly did not increase INHBA expression (Fig. 6I, J). Moreover, EdU assays and Transwell assays showed that circ-mut-miR and circ-mut-Δ690-760 still partially promoted the proliferation, migration, and invasion of AGS and HGC-27 cells, but circ-mut-miR + Δ690-760 had almost no cancer-promoting function compared with the empty vector. (Fig. 6K, L and Supplementary Fig. S5G–L). Taken together, our data suggest that circTHBS1 regulates INHBA expression in two coordinated mechanisms: as a sponge of miR-204-5p and a RBP decoy of HuR.

### CircTHBS1 exerts biological functions by targeting INHBA

**Fig. 7:**
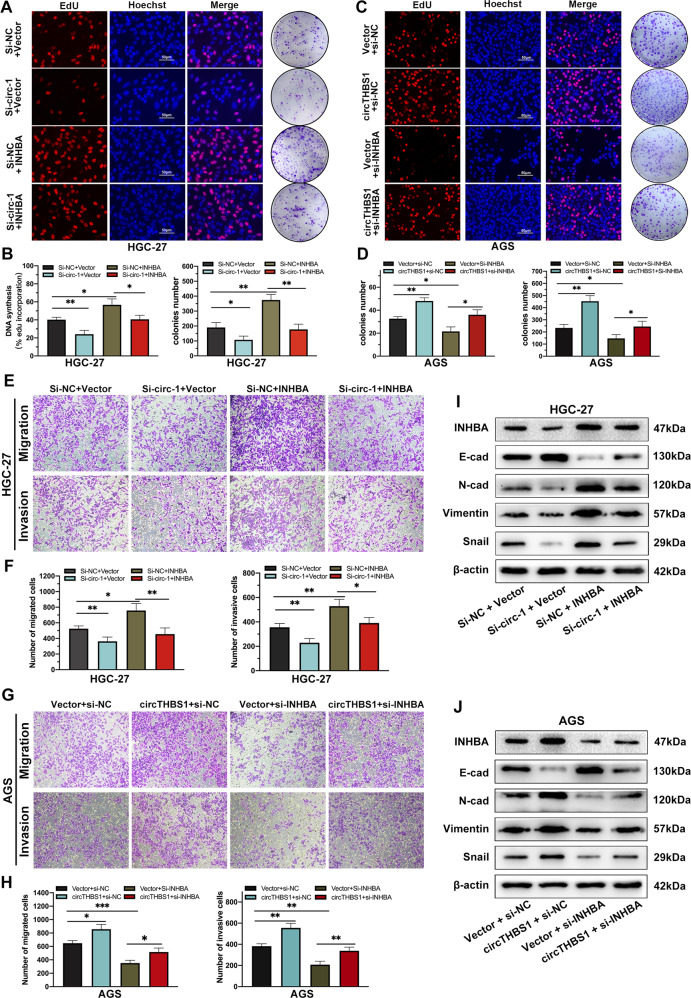
CircTHBS1 exerts biological functions by targeting INHBA. **A, B** EdU assays and colony formation assays revealed that co-transfection of INHBA overexpression plasmid and si-circTHBS1 in HGC-27 cells counteracted the suppressing effects caused by circTHBS1 knockdown. **C, D** EdU assays and colony formation assays showed that co-transfection of si-INHBA in circTHBS1 overexpression AGS cells counteracted the facilitative effect of circTHBS1 overexpression. **E, F** Transwell assays in HGC-27 cells showed that the up-regulation of INHBA restored the suppressive effect of circTHBS1 knockdown on cell migration and invasion. **G, H** The promotive effect on cell migration and invasion caused by circTHBS1 overexpression was weaken by INHBA knockdown in AGS cells. **I, J** Overexpression of INHBA reversed the effect of circTHBS1 knockdown on expression of key proteins involved in EMT. Quantitative data presented as the mean ± SD. *P < 0.05, **P < 0.01, ***P < 0.001 (Student’s *t*-test).

To explore whether the cancer-promoting function of circTHBS1 in GC cells were mediated by enhancing the expression of INHBA, rescue experiments were performed. Functional assays showed that circTHBS1 knockdown inhibited proliferation, migration, and invasion of AGS and HGC-27 cells, but this inhibition could be rescued by overexpression of INHBA (Fig. 7A, B, E, F and Supplementary Fig. S6A, B, E, F). In contrast, circTHBS1 overexpression promoted the proliferation, migration, and invasion of GC cells, but this promotive effect was reversed by INHBA silencing (Fig. 7C, D, G, H and Supplementary Fig. S6C, D, G, H). The results of western blot showed that circTHBS1 knockdown increased E-cadherin levels but decreased expression of mesenchymal cell markers (N-cadherin, Vimentin) and related transcription factors (Snail). And these circTHBS1 knockdown-induced effects were increased after INHBA reconstitution (Fig. 7I and Supplementary Fig. S6I). In contrast, INHBA knockdown counteracted the effect of circTHBS1 overexpression on the expression level of mesenchymal cell markers in AGS and HGC-27 cells (Fig. 7J and Supplementary Fig. S6J). In summary, circTHBS1 acts as an oncogenic factor in GC cells via targeting INHBA.

### CircTHBS1 promotes the growth and metastasis of GC in vivo

**Fig. 8:**
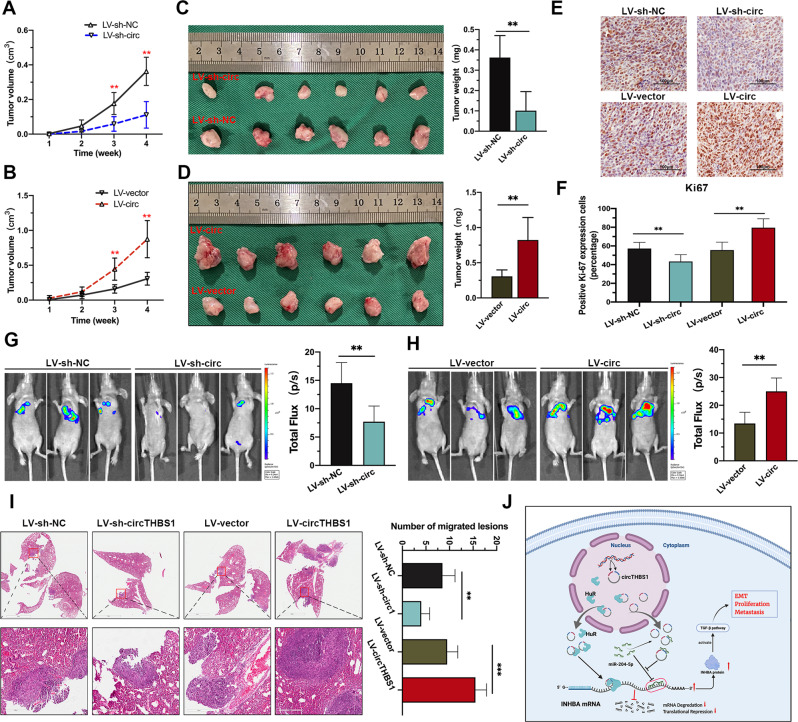
CircTHBS1 promotes the growth and metastasis of GC in vivo. **A, B** The growth curves of subcutaneous tumors in mice (*n* = 6), and the volume measured once a week. LV-sh-circ and LV-circ represent HGC-27 cells with stable knockdown and overexpression of circTHBS1, respectively. **C, D** Mice were sacrificed 4 weeks after subcutaneously injection with HGC-27 cells transfected with circTHBS1 overexpressed or knockdown lentivirus, and the volumes (left) and weights (right) of xenograft tumors were measured. **E, F** Immunohistochemical staining of Ki67 in xenograft tumors. **G, H** IVIS of mice 4 weeks after tail intravenous injection with HGC-27 cells. The luciferase intensities from the thoracic cavity were detected. **I** The metastatic nodules in the lungs of four groups of nude mice were stained with H&E. **J** The schematic diagram illustrating the mechanism of oncogenic function of circTHBS1. Quantitative data presented as the mean ± SD. *P < 0.05, **P < 0.01, ***P < 0.001 (Student’s *t*-test).

To evaluate the biological function of circTHBS1 in vivo, HGC-27 cells with stable circTHBS1 knockdown or overexpression were subcutaneously injected into the lateral abdomen of nude mice. Subcutaneous tumors were measured weekly (Fig. 8A, B) and harvested at week four. As shown in Fig. 8C and D, overexpression of circTHBS1 increased the weights of the harvested tumors compared to controls, while circTHBS1 knockdown decreased their weights. Immunohistochemical staining of Ki67 revealed that the proliferation index was significantly increased in the circTHBS1 overexpression group, while the circTHBS1 knockdown group showed the opposite trend (Fig. 8E, F). We further examined the effects of circTHBS1 on tumor metastasis using an in vivo imaging system (IVIS). The results suggested that circTHBS1 knockdown significantly inhibited lung metastasis compared to controls, while overexpression of circTHBS1 promoted lung metastases (Fig. 8G, H). Hematoxylin and eosin staining (H&E) of lung metastases confirmed that the numbers and sizes of metastatic nodules in the lungs were positively correlated with circTHBS1 expression (Fig. 8I). Based on these results, we concluded that circTHBS1 promotes GC proliferation and metastasis in vivo.

## Discussion

CircRNA is a special type of endogenous noncoding RNA with great abundance, high stability, and tissue-specific expression patterns. In this study, we found only a hand of circRNAs with regulatory potential. This is consist with previous report that most circRNAs are “junk” of mRNAs derived from splicing errors [19–21]. Thus, finding circRNAs with regulatory functions is a prerequisite for translating them into clinically effective therapeutic targets. However, the expression aberration may not be equivalent to their functional differences. In our study, ceRNA arrays were performed on 24 paired GC tissues and paracancerous tissues, and then self-organizing maps (SOM) algorithm was applied to analyze the data. As a neural network-based data matrix and visualization method, SOM first obtains a set of centroids (codebook vectors) and further maps each object in the dataset to the corresponding centroids with the principle of most similarity, and finally the most relevant genes are clustered together. In this study, 185 circRNAs with key regulatory potential were identified with SOM analysis, and an oncogenic circRNA, circTHBS1, was efficiently chosen for further study. After a series of experiments, we verified that circTHBS1 regulates the expression of INHBA by interacting with miR-204-5p and HuR (Fig. 8J), which is consistent with the results obtained from SOM analysis.

To date, most studies of circRNAs have shown that circRNAs perform their biological functions in three main ways: competing endogenous RNA, binding to RBPs, and translation. The ceRNA regulatory network mainly refers to non-coding RNAs competing to bind the same miRNA through the miRNA Response Element (MRE) so as to weaken their regulatory functions [8]. In this study, our results confirmed that circTHBS1 and INHBA share the same MRE in miR-204-5p, a downregulated miRNA with cancer suppressing function that was clearly validated in our previous study [7], therefore forming a circTHBS1/miR-204-5p/INHBA axis. Interestingly, we found that circTHBS1 overexpression plasmids with mutations in the miR-204-5p binding site still partially upregulated INHBA expression and promoted GC progression compared to the wild-type circTHBS1 plasmids. These observations encouraged us to hypothesize that circTHBS1 may regulate INHBA expression through other ways. RBPs serve as essential regulators in transcription and translation [22], and they have been reported to be involved in the regulation of downstream genes by interacting with circRNAs [23]. Moreover, circRNAs with dual-faceted regulation pathway (ceRNA and RBP) was identified in recent years [24–26]. For example, high expression of circSPARC in colon cancer contributed to accumulation of pro-phosphorylated (p)-STAT3 and facilitated its nuclear translocation via sponging miR-485-3p and recruitment of FUS. In our study, we found that cicTHBS1 physically interacted with HuR protein. As a conserved mRNA stability regulator [27], HuR contains many post-translational modifications sites, which make it easy to transport from nucleus to cytoplasm [28, 29]. HuR protein is highly expressed in most malignant cells and, more importantly, the cytoplasmic HuR accumulation in esophageal cancer, non-small cell lung cancer, meningioma, and GC predicts an unfavorable outcome [30]. Thus, it is hypothesized that translocation of HuR to the cytoplasm is necessary for it to promote mRNA stability. Abdelmohsen et al. have found that HuR can interact with several circRNAs [17]. Chen et al. reported that the highly expressed circAGO2 combined with HuR and promote latter’s transportation from nucleus to cytoplasm [26]. However, whether HuR is involved in the carcinogenesis and progression of GC remains unclear. In this study, we revealed that circTHBS1 facilitates HuR cytoplasmic accumulating, thereby enhancing the stability of INHBA mRNA.

Inhibinβ A (INHBA) is a member of the TGF-β superfamily that has multiple biological functions, including the promotion of tumor progression [31–33]. It was reported to activate the TGF-β signaling pathway by binding to the type II receptor (ActRII) and then heterodimerizing the type I receptor (ActRI/ALK4 or ActRI/ALK2) [34]. Elevated expression of TGF-β and its activated downstream molecules have been observed in many cancers, including GC [35, 36]. Our study confirmed that INHBA is the major participator of circTHBS1 in regulating GC cells proliferation, migration, and invasion. Additionally, elevated INHBA expression caused by circTHBS1 overexpression also enhanced the EMT of GC cells, which was consistent with the previous reports that the activation of TGF-β pathway can induce EMT [37–39]. To date, studies on the molecular mechanisms underlying the aberrant expression of INHBA in GC cells were poorly reported. Christopher W. Seder et al. reported that the expression of INHBA was affected by the demethylation and histone acetylation of its promoter [40]. In this study, we found that circTHBS1 upregulates INHBA expression by sponging miR-204-5p and recruiting HuR. This dual regulatory mechanism may be an important reason for the abnormal expression of INHBA. However, the regulation between genes is multilayered, multidirectional and interactive. Whether circTHBS1 influences INHBA expression in other ways remains to be further explored.

In conclusion, our study first demonstrates that circTHBS1 drives GC progression by increasing INHBA mRNA expression and stability in a ceRNA- and RBP-dependent manner, and its expression is significantly correlated with poor prognosis of GC patients. These findings indicated that circTHBS1 might be a prognostic biomarker and promising therapeutic target for GC.

## Supplementary information

Supplementary material

Supplementary Table S1

Supplementary Table S2

Supplementary Table S3

checklist

## Data Availability

The datasets supporting the conclusions of this article are included within the article and its Additional files.
