# Supplementary material for: CircTHBS1 drives gastric cancer progression by increasing INHBA mRNA expression and stability in a ceRNA- and RBP-dependent manner
Source: Cell Death Dis. 2022 Mar 25;13(3):266. doi: 10.1038/s41419-022-04720-0 (PMC8949653; doi:10.1038/s41419-022-04720-0)
Supplement: Supplementary file 1 — Supplementary material [file 41419_2022_4720_MOESM1_ESM.pdf]

Supplementary Figure S1

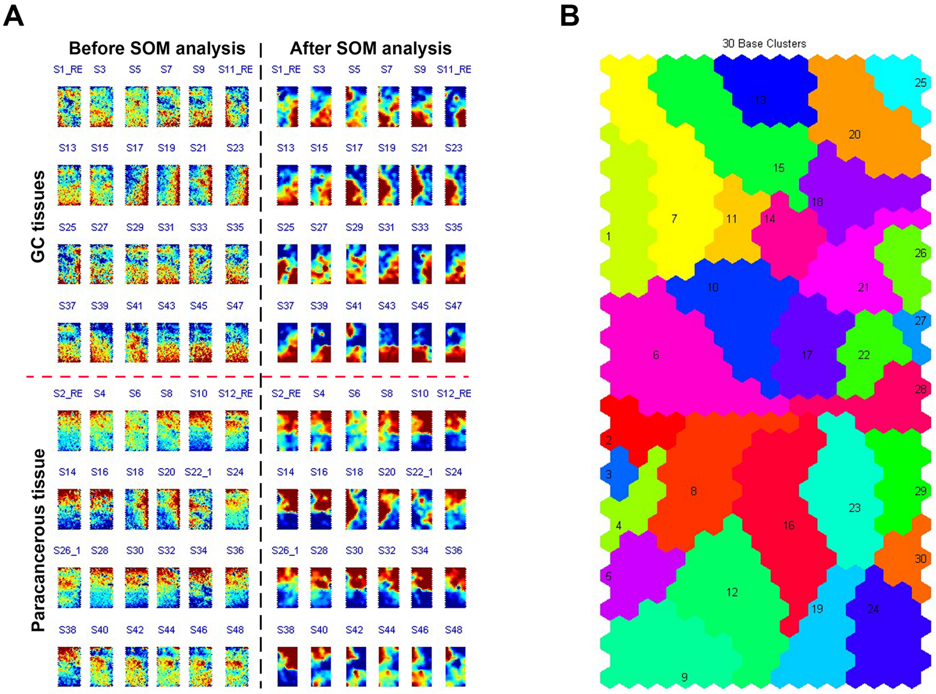

A. Self-Organizing map network exerts clustering function after unsupervised neural network training with data from ceRNA array, and then genes with similar properties of expression are clustered together. **B.** In the classification phase of SOM analysis, the neural network identifies the gene expression properties and eventually circRNAs and mRNAs expression patterns are classified into 30 clusters.

Supplementary Figure S2

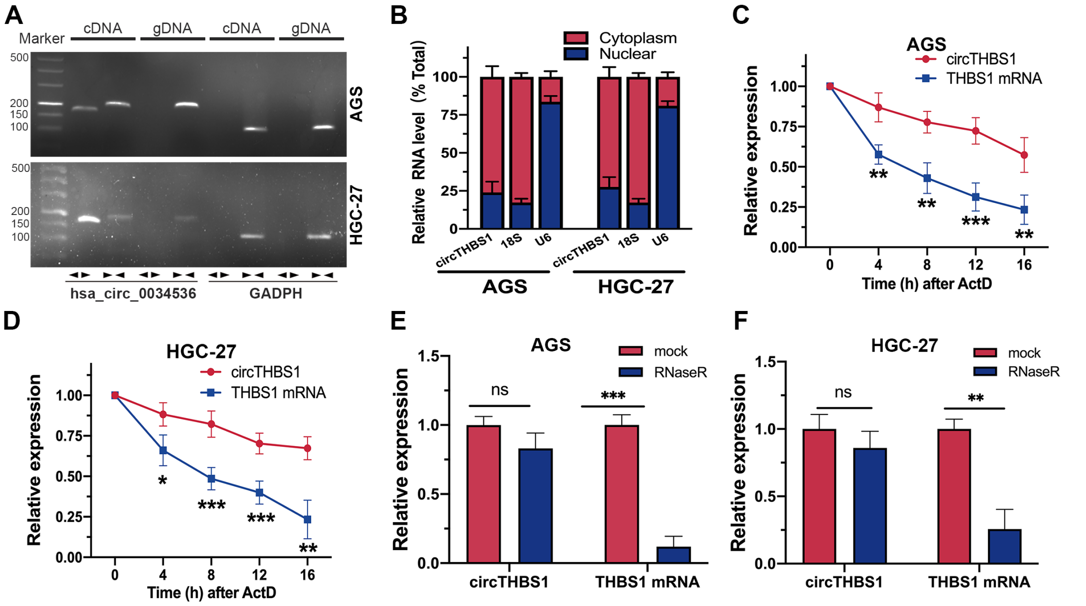

A. PCR and arose gel electrophoresis of products amplified respectively with convergent and divergent primers of circTHBS1 and GAPDH. **B.** qRT-PCR of Nuclear-cytoplasmic fractionation indicated that circTHBS1 mainly localized in the cytoplasm in AGS and HGC-27 cell lines. **C.** Actinomycin D treatment was used to evaluate the stability of circTHBS1 and THBS1 mRNA in AGS and HGC-27 cells. **D.** The expression of circTHBS1 and linear THBS1 mRNA in GC cells were detected by qRT-PCR after RNase R treatment.

Quantitative data presented as the mean ± SD. *P < 0.05, **P < 0.01, ***P < 0.001 (Student’s t-test).

Supplementary Figure S3

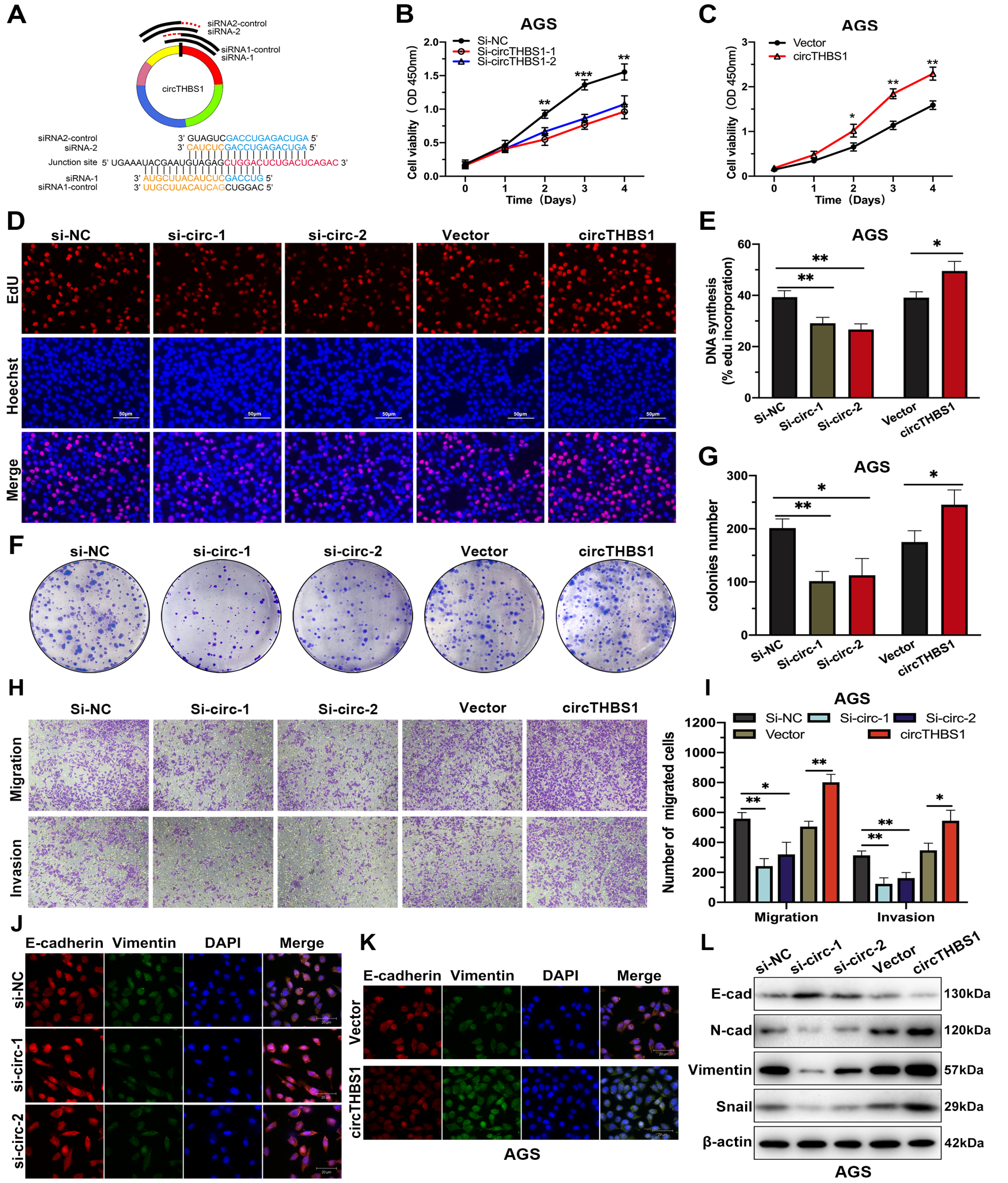

A. The diagram about the design of the small interfering RNAs of circTHBS1. **B, C.** The Effect of circTHBS1 on the proliferation of AGS cells detected by CCK-8. **D, E.** The Effect of circTHBS1 on the proliferation of AGS cells detected by EdU assay. Scale bar =50 μm. **F, G.** Colony formation assays to detect the effect of circTHBS1 expression on the proliferation of AGS cells. **H, I.** The effect of circTHBS1 expression on the migration and invasion of AGS measured by Transwell. **J, K.** The expression of E-cadherin (red) and vimentin (green) in AGS cells detected by Immunofluorescent staining, and DAPI (blue) was used for nuclear staining. Original magnification=400×; scale bar=20μm. **L.** Expression of EMT-related proteins in AGS cells were detected by Western blot; β-actin was used as an internal control. Quantitative data presented as the mean ± SD. *P < 0.05, **P < 0.01, ***P < 0.001 (Student's t-test).

Supplementary Figure S4

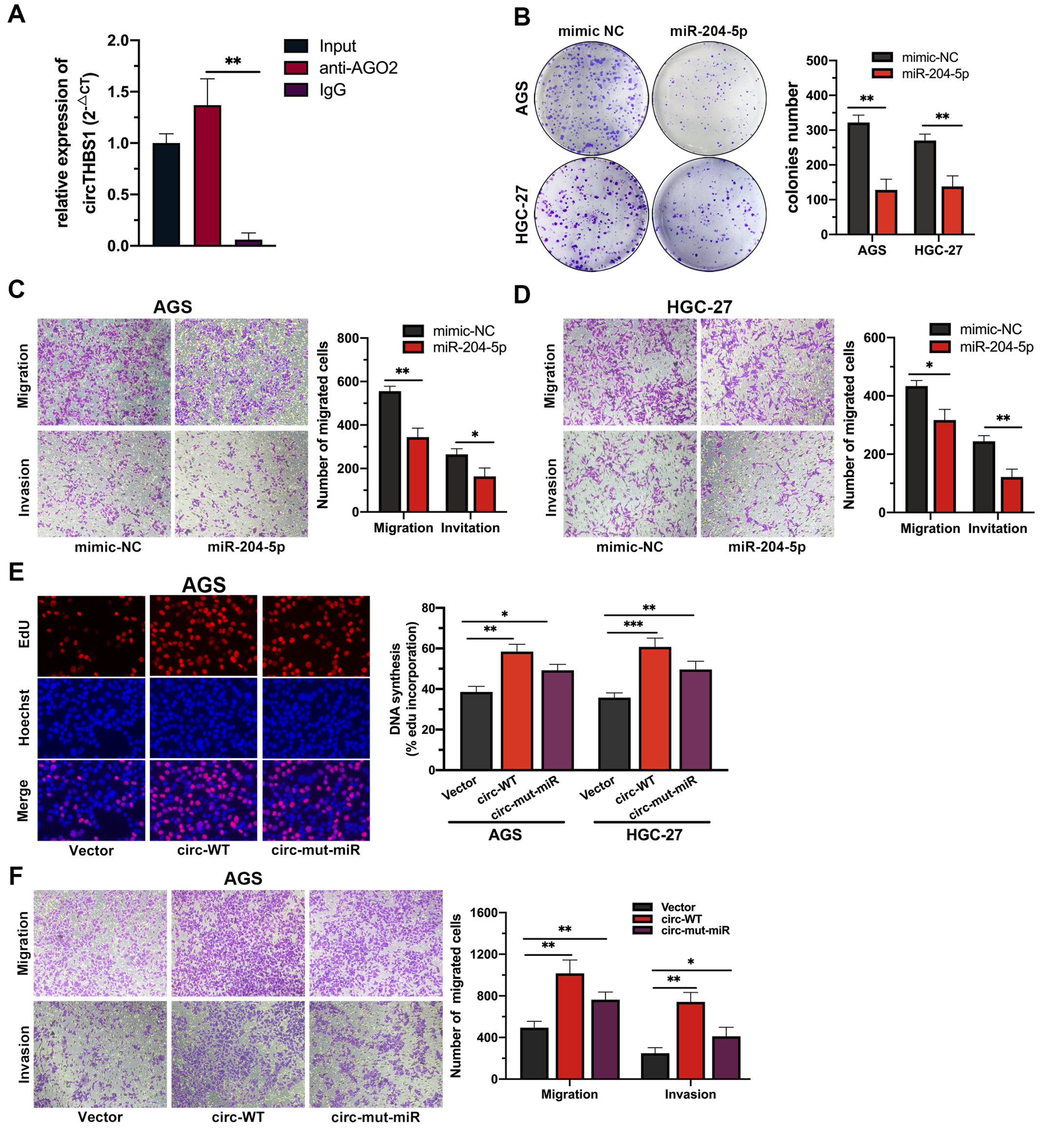

A. Level of circTHBS1 detected by qRT-PCR after RIP with anti-AGO2 in AGS cells. **B-D.** Colony formation assays and transwell assays were used to evaluate the effect of miR-204-5p on the proliferation, migration, and invasion of AGS and HGC-27 cells. **E,F.** EdU assays and transwell assays in AGS cells showed the partially promotive effect of circ-mut-miR (the binding site of miR-204-5p was mutated) on proliferation, migration, and invasion. Quantitative data presented as the mean ± SD. *P < 0.05, **P < 0.01, ***P < 0.001 (Student's t-test).

Supplementary Figure S5

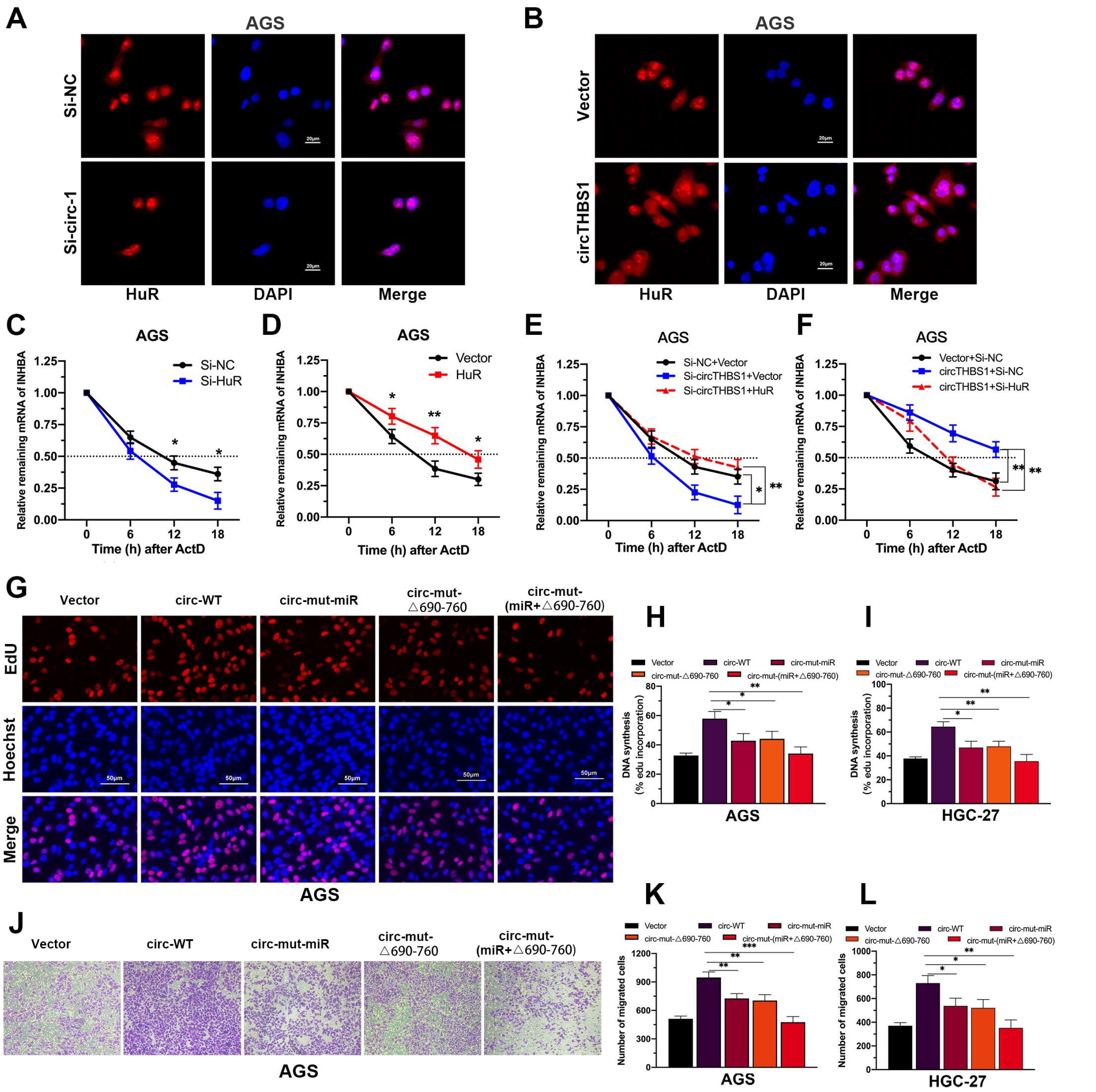

A, B. Immunofluorescent of HuR (red) in AGS cells after knockdown or overexpressing circTHBS1. Original magnification=400×; scale bar=20 μm. **C, D.** Degradation rates of INHBA mRNA were detected by qRT-PCR in AGS cells at different time periods. **E, F.** Degradation rate of INHBA mRNA in AGS cells transfected with different plasmids or small interferences. **G, H.** EdU assays of AGS cells treated with wild-type or mutant circTHBS1 overexpression plasmids. circ-mut-miR indicated the miR-204-5p binding site was mutant, circ-mut-△690-760 referred to the sequence from 690 to 760 nt of circTHBS1 was truncated. circ-mut-(miR+△690-760) referred to both mutated miRNA binding site and truncated part of the sequence. **I.** Statistical graphs of EdU assays in HGC-27 cells transfected with wild-type or mutant circTHBS1 overexpression plasmids. **J, k.** Transwell assays of AGS cells treated with wild-type or mutant circTHBS1 overexpression plasmids. **L.** Statistical graphs of transwell assays in HGC-27 cells transfected with wild-type or mutant circTHBS1 overexpression plasmids. Quantitative data presented as the mean ± SD. *P < 0.05, **P < 0.01, ***P < 0.001 (Student's t-test).

Supplementary Figure S6

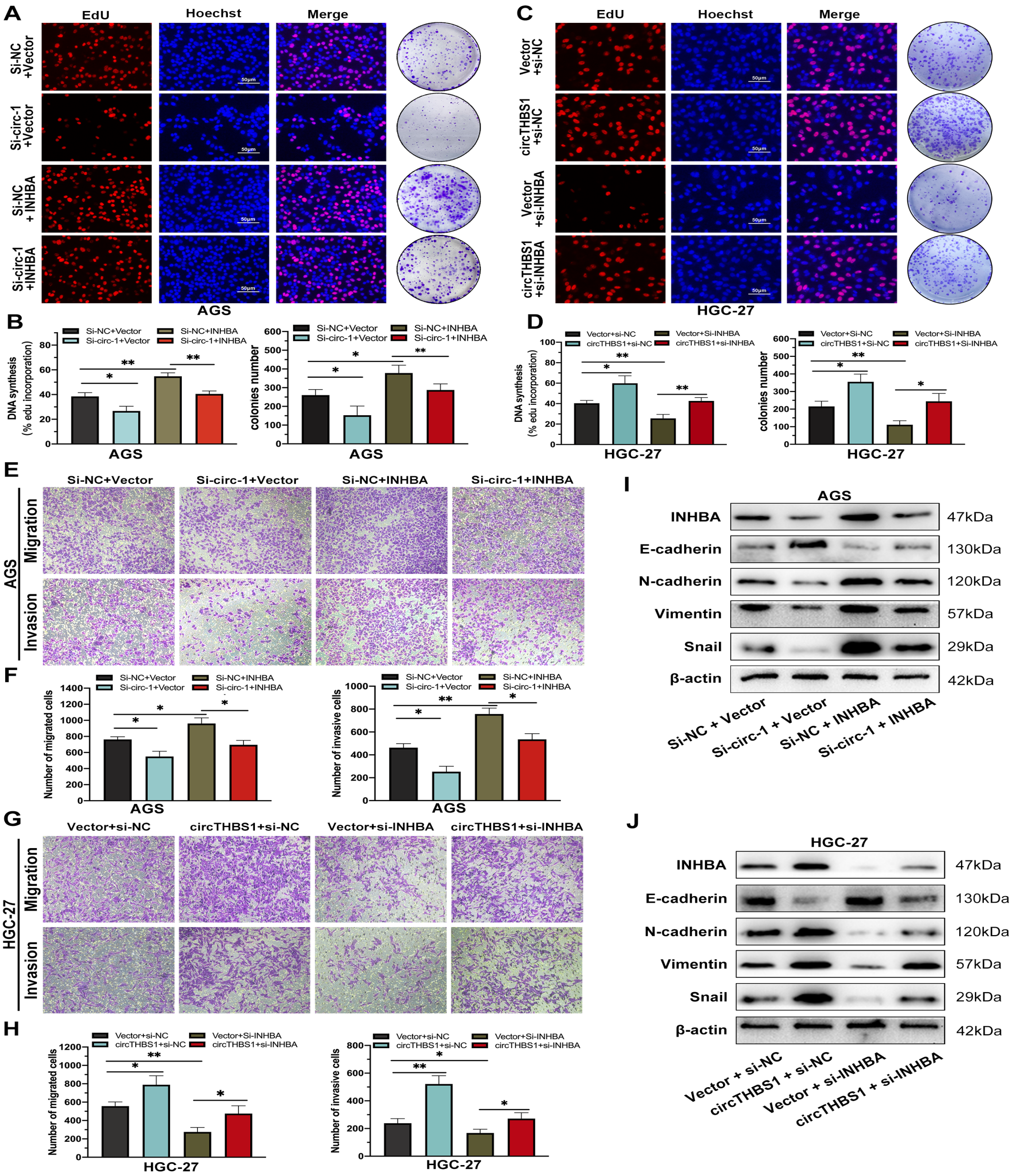

A, B. EdU assays and colony formation assays suggested that co-transfection of INHBA overexpression plasmids and si-circTHBS1 in AGS cells counteracted the inhibitory function caused by circTHBS1 knockdown. **C, D.** EdU assays and colony formation assays showed that knockdown of INHBA reversed the proliferation-promoting effect of circTHBS1 overexpression in HGC-27 cells. **E, F.** Transwell in AGS cells suggested that overexpression of INHBA restored the suppressive effect of circTHBS1 knockdown on migration and invasion. **G, H.** The promotive effect of circTHBS1 overexpression on migration and invasion of HGC-27 cells was impaired after INHBA knockdown. **I, J.** The results of Western blot showed that INHBA reversed the effect of circTHBS1 knockdown on expression of key proteins involved in EMT. Quantitative data presented as the mean ± SD. *P < 0.05, **P < 0.01, ***P < 0.001 (Student's t-test).

Supplementary Figure S7

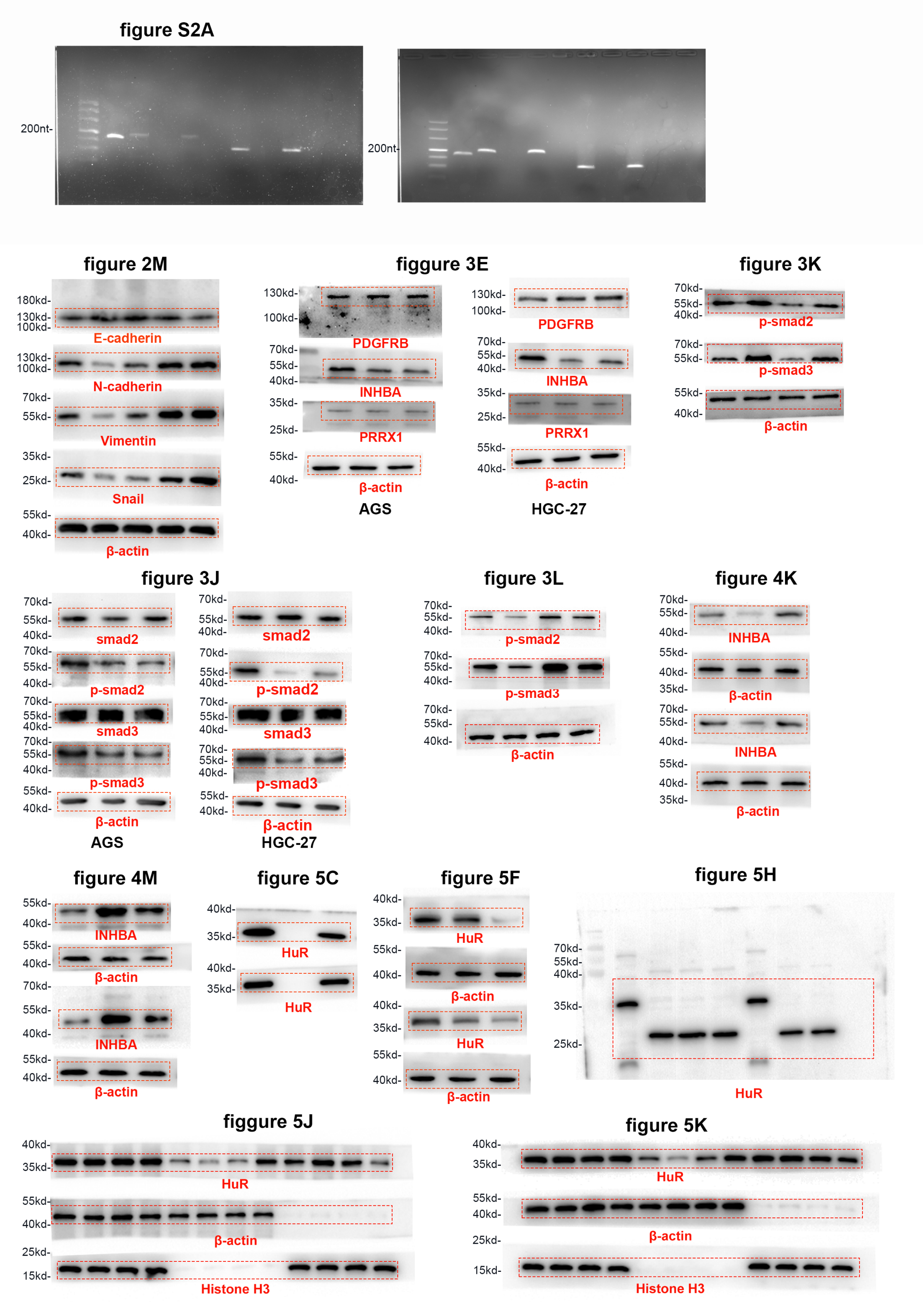

The uncropped scans of western blots and gels from the main figures

Supplementary Figure S8

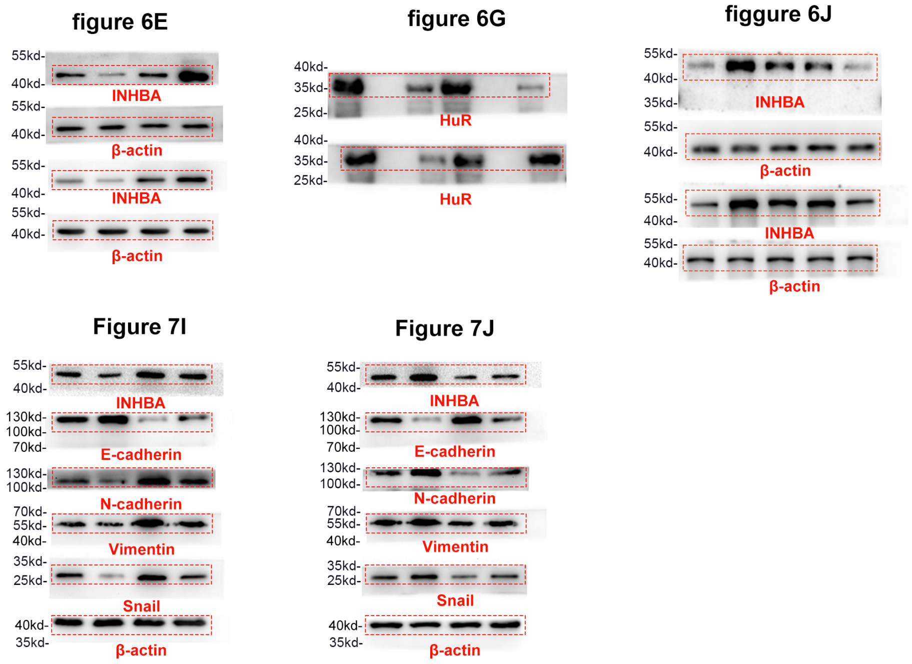

The uncropped scans of western blots from the main figures

Supplementary Table S4

Clinical information of 24 patients for ceRNA microarray sequencing

Sample number	TNM Stage	Tumor in the cardia	Gender	Age
S1-RE,S2-RE	T4bN0M0（IIIB）	Yes	Male	71
S3,S4	T2N0M0(Ib)	Yes	Male	64
S5,S6	T2N0M0(Ib)	Yes	Female	60
S7,S8	T2N0M0(IB)	No	Male	65
S9,S10	T2N0M0(IB)	No	Male	76
S11-RE,S12-RE	T4aN0M0(IIB)	No	Female	50
S13,S14	T1aN0M0（IA）	Yes	Male	58
S15,S16	T1aN0M0（IA）	Yes	Male	78
S17,S18	T1bN1M0(IB)	Yes	Female	73
S19,S20	T1bN0M0(IA)	No	Male	51
S21,22	T1bN0M0（IA）	No	Female	63
S23,24	T1bN0M0（IA）	No	Male	64
SS25,S26	T4aN2M0(IIIB)	Yes	Female	67
S27,S28	T4aN3bM0(IIIB)	Yes	Male	62
S29,S30	T4aN3bM0（IIIC）	Yes	Male	58
S31,S32	T4aN3M0(IIIc)	No	Female	63
S33,S34	T4aN3aM0(IIIC)	No	Male	66
S35,36	T4aN3aM0（IIIC）	No	Male	75
S37,S38	T4aN2M0（IIIC）	Yes	Female	72
S39,S40	T4aN2M0（IIIB）	Yes	Male	65
S41,S42	T4aN3aM0（IIIC）	Yes	Male	66
S43,S44	T4aN3aM0(IIIC)	No	Male	67
S45,S46	T4aN3aM0（IIIC）	No	Female	60
S47,S48	T4aN3aM0（IIIC）	No	Male	66

Note: Sample numbers with odd numbers represent tumor tissues; sample numbers with even numbers represent normal paracancerous tissues

Supplementary Table S5

Primer, probes, and siRNAs used in the experiments

Gene	Sequence (5'-3')	Application
hsa_circ_0034539	F: GCCAATACCTTTTTCTAGGAATGTG<br>R: CATCACCTCGACCATCGCTATAA	qRT-PCR
hsa_circ_0034536	F: CCTGAAATACGAATGTAGAGCTGG<br>R: CTTTGTCATGGTCAGCCTGGT	qRT-PCR
hsa_circ_0077046	F: CCTGGGATCATGCTGATGGG<br>R: TCCTGAAGTTCTGTTGGCGA	qRT-PCR
has_circ_0003251	F:CCACTAAAAGCAAAGGCAGCA<br>R: GGCCTTCTTTGACTTGAGAAACTG	qRT-PCR
has_circ_0011939	F: CACGACAGGTACCTACCAGGATT<br>R: ATCTCTGCTCCATTTAGCGTCTT	qRT-PCR
has_circ_0012363	F: GGTGCCAGTAACAATTCTGAAAC<br>R: CTTCCAACAAACGCTGAATCAA	qRT-PCR
β-actin	F:CCCCAGGCACCAGGGCGTGAT<br>R:GTCATCTTCTCGCGGTTGGCCTTGGGGT	qRT-PCR
GAPDH	F: AGAAGGCTGGGGCTCATTTG<br>R: AGGGGCCATCCACAGTCTTC	qRT-PCR
GAPDH (divergent)	F: TGTACCATCAATAAAGTACCCTGTG<br>R: AAATCCGTTGACTCCGACCT	qRT-PCR
U6	F: CTCGCTTCGGCAGCACA<br>R: AACGCTTCACGAATTTGCGT	qRT-PCR
18s	F: ACACGGACAGGATTGACAGA<br>R: GGACATCTAAGGGCATCACA	qRT-PCR
hsa-miR-204-5p	F: CCCATCGTTAAGCAATGCATGAC	qRT-PCR
hsa-miR-211-5p	F: GATGCTGTAATGGATGATATGA	qRT-PCR
hsa-miR-378a-3p	F: GCGCACTGGACTTGGAGTC	qRT-PCR
hsa-miR-422a	F: ACUGGACUUAGGGUCAGAAGGC	qRT-PCR
Universal	R: GCGAGCACAGAATTAATACGAC	qRT-PCR
INHBA	F: CCTCCCAAAGGATGTACCCAA<br>R: CTCTATCTCCACATACCCGTTCT	qRT-PCR
PDGFRB	F:AGCACCTTCGTTCTGACCTG<br>R:TATTCTCCCGTGTCTAGCCCA	qRT-PCR
PRRX1	F: CAGGCGGATGAGAACGTGG<br>R: AAAAGCATCAGGATAGTGTGTCC	qRT-PCR
HuR	F: GGGTGACATCGGGAGAACG<br>R: CTGAACAGGCTTCGTAACTCAT	qRT-PCR
si-circTHBS1	NC：ATGCGCTAGATGATCGACGG<br>si-1：AGTCAGAGTCCAGCTCTAC<br>si-2：GTCCAGCTCTACATTCGTA	siRNA
si-HuR	si-1：CAGUUUCAAUGGUCAUAAACC<br>si-2：CGAGCUCAGAGGUGAUCAAAG<br>si-3：CAGUUUCAAUGGUCAUAAACC	siRNA
si-INHBA	si-1：AGUUCUUUGUCAGUUUCAAGG<br>si-2：GGAAGACGCUGCACUUCGAGA<br>si-3：CCAUGUCCAUGUUGUACUAUG	siRNA
miR-204-5p mimics	sense: UUCCCUUUGUCAUCCUAUGCCU<br>antisen: AGGCAUAGGAUGACAAAGGGAA	mimics
mimics NC	CAGTACTTTTGTGTAGTACAAA	mimics
inhibitor	UCCAUCAUCAAAACAAAUGGAGU	inhibitor
circTHBS1 probe	NC probe：CAACTGACTCGTCTTGATCT-/3bio/<br>Probe1：GTCCAGCTCTACATTCGTAT-/3bio/<br>Probe2：AGTCCAGCTCTACATTCGTA-/3bio/	RNA-pull down
miR-204-5p probe	TCCATCATCAAAACAAATGGAGT	FISH
circTHBS1 probe	TGCTTACATCTCGACCTGAGACT	FISH

Supplementary Table S6

Antibodies used in the present study

Product	Source	No. of Catalogue
**Western blot:**		
**Primary antibody:**		
anti-β-Actin	Proteintech	66009-1-Ig
anti-N-Cadherin	Cell Signaling Technology	#13116
anti-E-Cadherin	Cell Signaling Technology	#14472
anti-Vimentin	Cell Signaling Technology	#5741
anti-Snail	Cell Signaling Technology	#3879
anti-smad2	Abcam	ab40855
anti-p-smad2	Abcam	ab53100
anti-smad3	Abcam	ab40845
anti-p-smad3	Abcam	ab52939
anti-PRRX1	Invitrogen	MA5-26580
anti-PDGFRB	Abcam	ab69506
anti-INHBA	Proteintech	10651-1-AP
anti-AKT	Cell Signaling Technology	#9272
anti-p-AKT	Cell Signaling Technology	#4060
anti-mTOR	Cell Signaling Technology	#2983
anti-p-mTOR	Cell Signaling Technology	#5536
anti-HuR	Abcam	ab200342
ant-Histome3	Proteintech	17168-1-AP
**Secondary antibody**		
anti-rabbit IgG-HRP	Proteintech	SA00001-15
anti-mouse IgG-HRP	Proteintech	SA00001-1
**IHC and IF:**		
**Primary antibody:**		
Anti-Ki67	Abcam	ab15580
anti-E-Cadherin	Cell Signaling Technology	#14472
anti-Vimentin	Cell Signaling Technology	#5741
**Secondary antibody**		
Goat Anti-Mouse IgG H&L (Alexa Fluor® 647)	Abcam	Ab150115
Goat Anti-Rabbit IgG H&L (Alexa Fluor® 594)	Abcam	Ab150080
**RIP:**		
anti-Argonaute-2	Abcam	ab186733
anti-HuR	Abcam	ab200342
IgG	Abcam	ab172730

English Editing Certificate

AJE®

Editing Certificate

This document certifies that the manuscript

CircTHBS1 drives gastric cancer progression by increasing INHBA mRNA expression and stability in a ceRNA- and RBP-dependent manner

prepared by the authors

Shengkui Qiu1,2,*, Bowen Li1,*, Yiwen Xia1*, Zhe Xuan1,*,Zheng Li1,*, Li Xie1, Chao Gu1, Jialun Lv1, Chen Lu1, Tianlu Jiang1, Lang Fang1, Penghui Xu1, Jing Yang1, Ying Li1, Zetian Chen1, Lu Zhang1, Linjun Wang1, Diancai Zhang1, Hao Xu1, Weizhi Wang1,#, Zekuan Xu1,3,#

was edited for proper English language, grammar, punctuation, spelling, and overall style by one or more of the highly qualified native English speaking editors at AJE.

This certificate was issued on **July 12, 2021** and may be verified on the AJE website using the verification code **D873-13C5-39C5-D02B-26FB** .

Neither the research content nor the authors' intentions were altered in any way during the editing process. Documents receiving this certification should be English-ready for publication; however, the author has the ability to accept or reject our suggestions and changes. To verify the final AJE edited version, please visit our verification page at aje.com/certificate. If you have any questions or concerns about this edited document, please contact AJE at support@aje.com.

AJE provides a range of editing, translation, and manuscript services for researchers and publishers around the world. For more information about our company, services, and partner discounts, please visit aje.com.
